# Hypertension related toxicity of chloroquine explains its failure against COVID-19: Based on rat model

**DOI:** 10.3389/fphar.2022.1051694

**Published:** 2022-11-30

**Authors:** Junqi Wang, Xian Jing, Lizhong Hua, Yuling Zheng, Shiheng Hu, Jing Xiao, Dawei Guo, Wenda Wu, Hui Ji, Lin Peng, Shanxiang Jiang, Xiuge Gao

**Affiliations:** ^1^ MOE Joint International Research Laboratory of Animal Health and Food Safety, College of Veterinary Medicine, Nanjing Agricultural University, Nanjing, China; ^2^ Engineering Center of Innovative Veterinary Drugs, Center for Veterinary Drug Research and Evaluation, Nanjing Agricultural University, Nanjing, China; ^3^ School of Animal Husbandry and Veterinary Medicine, Jiangsu Vocational College of Agriculture and Forestry, Jurong, Jiangsu, China

**Keywords:** COVID-19, safety evaluation, antiviral drug, toxicology, chloroquine, hypertension

## Abstract

Chloroquine was once thought to be a promising treatment for COVID-19 but it quickly failed due to its inefficiency and association with increased mortality. Further, comorbidities such as hypertension may have contributed this failure. The safety and toxicity of chloroquine at doses required for treating SARS-CoV-2 infection in hypertensive patients remain unknown. Herein, to investigate these effects, we performed a safety evaluation of chloroquine at the approved dose (63 mg/kg) and at a high dose (126 mg/kg) in hypertensive rats. We found that chloroquine increased the mortality of hypertensive rats to 18.2% and 100%, respectively, after 7 days. During the chloroquine exposure period, the bodyweight, feed, and water consumption of hypertensive rats were decreased significantly. In addition, we show that chloroquine induces prolongation of QTc interval, elevation of LDH and CK, and histopathological damage of the myocardium in hypertensive rats. Ocular toxicity was observed in hypertensive rats in the form of hemorrhage in the eyes and retinal damage. Furthermore, we also observed intestinal toxicity in hypertensive rats, which presented as thinning intestinal walls with hemorrhagic contents, and histopathological changes of the jejunum. Hepatotoxicity was also evidenced by elevated ALT, and vacuolization of hepatocytes was also observed. Nephrotoxicity was observed only in high dose chloroquine-treated hypertensive rats, presenting as alterations of urinalysis and renal function. Immune alterations were also found in high-dose chloroquine-treated hypertensive rats with elevation of serum IL-10, IL-1β and GRO, and moderate damage to the spleen. In summary, this study partially explains the reason for the failure of chloroquine as a COVID-19 therapy, and underlines the importance of safety evaluation and medical supervision of chloroquine to avoid patient harm, especially to those with hypertension.

## Introduction

Chloroquine (CQ), a 4-aminoquinoline drug, has long been extensively used for controlling malaria and certain autoimmune diseases, including rheumatoid arthritis and systemic lupus erythematosus (Slater, 1993). Since the emergence of widespread resistance in *Plasmodium falciparum* and CQ-induced acute poisoning and death, the market value of CQ has declined, leading to its replacement with its analogue, hydroxychloroquine, which is safer and much more efficacious (Nirk et al., 2020). Recently, CQ has gained increased global attention due to its initial promise as a potential antiviral agent for combating SARS-CoV-2 in early preclinical studies (Huang et al., 2020; Multicenter collaboration group of Department of Science and Technology of Guangdong Province and Health Commission of Guangdong Province for chloroquine in the treatment of novel coronavirus pneumonia, 2020). Given the absence of specially approved drugs for use in COVID-19 cases and the impact of the ongoing COVID-19 pandemic, these exciting findings on the *in vitro* efficacy of CQ and hydroxychloroquine against SARS-CoV-2, along with preliminary clinical observations, prompted clinical trials and the emergency use of chloroquine worldwide (U.S. Food & Drug Administration, 2020; European Medicines Agency, 2020). Since then, numerous clinical trials of CQ and hydroxychloroquine, at various doses and regimens, were carried out during the fight against COVID-19 (Axfors et al., 2021). CQ and hydroxychloroquine were subsequently approved for emergency use in hospitalized COVID-19 patients by the Food and Drug Administration (FDA) and the European Medicines Agency (EMA) early on in the pandemic (U.S. Food & Drug Administration, 2020; European Medicines Agency, 2020). In China, CQ was officially recommended as a clinical trial drug for treating COVID-19 in the sixth edition of the Diagnosis and Treatment Program of COVID-19 (National Health Commission of the People’s Republic of China, 2020). However, the promise of CQ and hydroxychloroquine faded quickly following disappointing outcomes from large randomized clinical trials, as confirmed by the World Health Organization (Consortium et al., 2021). The WHO declared that the failure of CQ/hydroxychloroquine against COVID-19 was due to invalid anti-SARS-CoV-2 effects, along with multiple side effects and increased mortality (Boulware et al., 2020; Axfors et al., 2021). Subsequently, all clinical trials and the emergency use of CQ for treating COVID-19 were completely halted in most countries based on these findings, which showed no benefits of CQ/hydroxychloroquine treatment and increased potential risks (Consortium et al., 2021). However, treatment of COVID-19 with CQ continued in some countries, including China (National Health Commission of the People’s Republic of China, 2021) and India (Dinesh et al., 2021). Various publications have summarized and discussed the controversial journey of CQ/hydroxychloroquine (Das et al., 2021; Megarbane, 2021; Shah, 2021). However, the exact reasons underlying its failure against COVID-19 remain unclear; they may involve the narrow window between the CQ dosages related to toxicity and efficacy against SARS-CoV-2 strains.

On one hand, CQ has been demonstrated incompatible results against SARS-CoV-2 in different *in vitro* models (Hoffmann et al., 2020), suggesting its uncertain antiviral effects in further clinical trials. On the other hand, the dose and regimens of CQ and hydroxychloroquine were substantially higher than recommended in malaria or rheumatological conditions (Organization, 2015), leading to increased mortality and multiple side effects in hospitalized COVID-19 patients (Axfors et al., 2021). In addition, the physical health of some SARS-CoV-2-infected patients were very complicated because about 60–90% of hospitalized patients had comorbidities (Wiersinga, Rhodes, Cheng et al., 2020). This presented a challenge in terms of the safety evaluation of drugs used in clinical trials. Among these comorbidities, hypertension, one of the most common risk factors, has been linked to severe illness and mortality in COVID-19 (Zhou et al., 2020; Peng et al., 2021). A large-scale clinical trial including 5,700 individuals confirmed that COVID-19 patients hospitalized in the United States considered hypertension (56.6%) as the most common comorbidity (Rosenberg et al., 2020). Similar high rates of hypertension in hospitalized COVID-19 patients were found in clinical trials carried out in other countries, including, but not limited to China and Italy (Grasselli et al., 2020; Zhou et al., 2020). Importantly, hypertension alters metabolic status and SARS-CoV-2 infection damages multiple organs, especially the liver and lungs, thereby presenting a challenge for further safety evaluation of experimental anti-COVID-19 drugs in the clinic. Therefore, we hypothesized that hypertension may affect the determination of the safety and efficacy of CQ in clinical trials for treating COVID-19. In addition, CQ has been extensively used for suicide since the 1980s in France and Zimbabwe, among others (Riou et al. Baud, 1988; Clemessy et al., 1996; Ball et al. Nhachi, 2002). Unfortunately, CQ overdose has been the cause a large number of deaths. Ingestion of CQ in overdose induces hypotension, arrhythmias, acute dyspnea, coma, and fatal cardiac arrest, which are lethal risk factors during CQ self-poisoning. Taken together and considering that hypertension is one of the most common basic diseases in the elderly population, which is also the population that is most affected by severe COVID-19, this raising the question of whether CQ is safe to hypertensive individuals in clinical trials. Therefore, it is critically important to elucidate the potential toxic effects of CQ in models of hypertension. Moreover, such an investigation will also further accelerate the repurposing process of CQ for treatment of coronavirus related diseases.

In this study, to avoid the harmful effects induced by the irrational use of CQ in the general population, especially during the COVID-19 pandemic, and to further understand the potential toxic effects of CQ in hypertensive patients, we sought to investigate the toxicity of CQ by employing hypertensive rats due to animal disease model is one of the most efficient approaches to reveal potential toxicity and health risk of chemicals, which will further help clinical pharmacists to recognize and avoid drug-induced risks especially the drugs on trial. Therefore, the present work focused on three aspects: 1) evaluating the safety of CQ in hypertensive rats using equivalent doses for anti-SARS-CoV-2 treatment; 2) understanding the toxic effects of CQ on hypertensive rats; 3) trying to elucidate the reason behind CQ toxicity and subsequent failure during the COVID-19 pandemic.

## Materials and methods

### Reagents and drugs

Nω-Nitro-L-arginine methyl ester hydrochloride (L-NAME, Sigma-Aldrich, N5751, St. Louis Missouri, United States), chloroquine phosphate (CQ, Aladdin, C129284, Shanghai, China), diethyl ether (Sinopharm Chemical Reagent, 10009318, analytical reagent, Shanghai, China), 4% paraformaldehyde (Biosharp, BL539A, Hefei, China), 0.9% (w/v) sodium chloride (Guojing Pharmaceutical Co., Ltd, Lishui, Zhejiang, China). Chloroquine phosphate was freshly prepared before use.

### Animals

Male specific pathogen free (SPF) Wistar rats (N = 80, 5 weeks old, 200–250 g) were purchased from Charles River (Beijing Vital River Laboratory Animal Technology Co., Ltd., Beijing, China). All rats were reared in rooms of the experimental animal center of Nanjing Agricultural University. Three or four rats were kept per cage in the barrier system with constant-humidity (55% ± 5%), constant-temperature (24°C ± 1°C), and a natural 12 h/12 h light-dark cycle. Each rat was provided with sufficient diet obtained from Jiangsu Xietong, Inc. (Nanjing, China) and clean drinking water. The detailed composition of diet for all rats in this study is in accord with the formulation of AIN-93G (Reeves et al., 1993). The animal protocols carried out in this work were evaluated and approved by the Committee on Animal Welfare and Ethics of Nanjing Agricultural University (No.20210514069) in accordance with the Regulations on the Administration of Laboratory Animals in China.

### Hypertensive rat model

A hypertensive rat model was established according to previous published protocols with minor alteration (Adaramoye et al., 2012; Lerman et al., 2019). Briefly, after a week of acclimatization, sixty 6-week-old rats (250 g ± 20 g) were randomly divided into two groups, namely, the normal control group (n = 10), and the model group (*n* = 50). Rats in the model group were treated by intraperitoneal (i.p.) injection of the non-specific NOS inhibitor, L-NAME, at a dose of 20 mg/kg twice daily for 6 weeks (Rees et al. Moncada, 1990; Bader, 2010). All rats in the control group received an equal volume of saline *via* i.p. injection. During the hypertension modeling period, rats were fed with the standard diet and clean tip water in the experimental environment. Rat tail artery pressure was measured by performing a non-invasive blood pressure measurement and analysis system (BP 2000, Softron, Beijing, China) at a fixed time every week. The systolic blood pressure (SBP) and the diastolic blood pressure (DBP) of rats in an awake and quiet state were recorded using a computerized data acquisition system (Softron, Beijing, China). SBP and DBP measurements were conducted three or four times to obtain average value. After administration of L-NAME for 6 weeks, rats with SBP (>160 mmHg) and DBP (100 mmHg) were used for subsequent studies. In addition, to avoid recovery of blood pressure, a low dose of L-NAME (10 mg/kg, once daily) was injected once daily in the morning 2 h before administration of CQ.

### Dosage regimen of CQ in hypertensive rats

**FIGURE 1:**
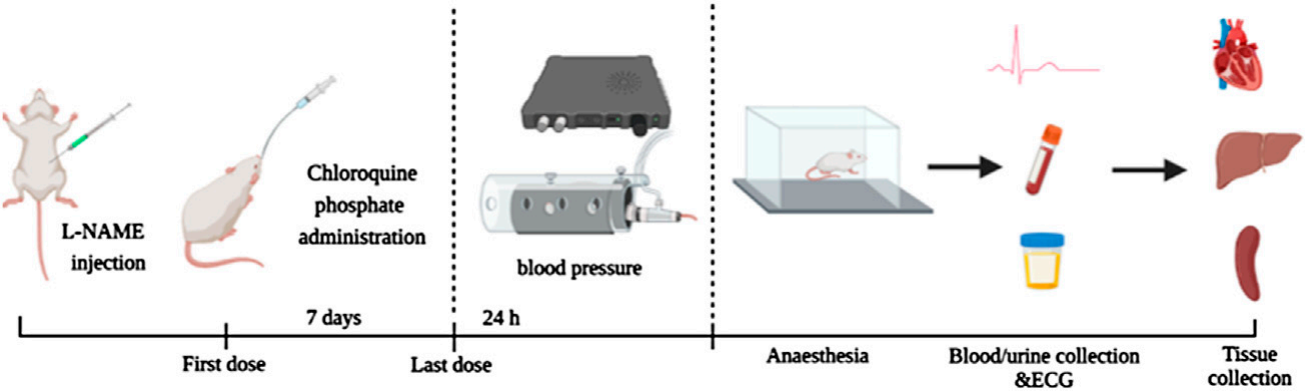
Schematic diagram of animal experiment.

The hypertensive rats were randomly divided into three groups: 1) saline (*n* = 9, control group), 2) 63 mg/kg CQ (*n* = 11, equivalent to clinical dosage), 3) 126 mg/kg CQ (*n* = 12, high dose). These doses of CQ used in rats were equivalent to the human dose by calculation using the following formula: Animal equivalent dose (AED; mg/kg) = Human dose (HED; mg/kg) × Km ratio, where Km ratio = (Human Km / Animal Km), Km is estimated by dividing the average body weight (kg) of the species to its body surface area (m^2^) (Xu et al., 2002). Nine healthy rats were used as negative controls in the following experiments. CQ solution and saline were administered to rats by gavage twice daily for seven consecutive days. During the administration period of CQ, all rats were given a standard diet and clean water *ad libitum*. A schematic diagram of animal experiments is shown in Figure 1.

### Clinical observations

All rats were observed for any changes once daily during the acclimatization period and twice daily during the treatment period. Detailed clinical observations included behavior, reaction to treatment, or ill-health. Any abnormal conditions were recorded at the time in terms of nature and severity, date and time of onset, and duration. Observations for viability/mortality, food intake, and water consumption were recorded daily. Rats were weighed every 2 days. In addition, eye reactions of all rats were inspected visually twice daily after CQ administration.

### Blood pressure (BP) determination

To understand the effects of CQ on SBP and DBP in rats, tail-cuffing was carried out every 3 days as previously described. Briefly, SBP and DBP of rats in an awake and quiet state were measured three or four times to obtain average values used for further analysis. All rats were monitored until they were too weak to support the BP assessment.

### Electrocardiogram monitoring

In order to explore the effect of CQ on the heart rhythm of rats, we monitored the electrocardiogram on the eighth day. Five rats in each group were anesthetized with diethyl ether. After the rats were stabilized, ECG monitoring was carried out by performing BL-420N computerized bio-signal acquisition system (TaiMen, Chengdu, China) for at least 10 min according to previous report (Gao et al., 2022). After ECG of Rats in each group were collected, we analyzed the changes of rat cardiac cycle by statistical PR, QRS and QT intervals. The sudden deaths of rats received high-dose CQ on day 6 prevent us from performing ECG examinations at the pre-planned time. Unfortunately, rats in the approved and high dose of CQ group were extremely weak and could not support anaesthetization before ECG, making the urgent ECG monitoring on day 6.

### Biological sample collection and pretreatment

At the end of the CQ administration period, all rats except those in the high-dose CQ treatment group were fasted for 12 h before the collection of rat plasma, serum, urine and organic tissues. Importantly, samples from rats in the high-dose CQ group were emergency collected on day 6 due to poor health and alarming mortality of most rats in this group. Three rats in the high-dose CQ group and five rats in each other group were selected to use metabolic cages for urine collection, and urine test within 1 h of sample collection. Next, we collected at least five copies of whole blood from rats in each group *via* the jugular vein. Here, 200 μl of blood was obtained from each rat and transferred to a EDTA tube for hematological examination. Subsequently, 800 μl of blood was collected from each rat in a non-anticoagulant tube, which was then centrifuged at 1,000 × g at 4°C for 10 min to obtain serum. After centrifugation, we obtained rat serum of each group at least in triplicate used for biochemical analysis and cytokine determination. The collected rat blood plasma was stored at 4°C and analyzed within 4 h while serum was stored at −20°C until further biochemical assessment and cytokine analysis. Furthermore, rats were kindly sacrificed under general anesthesia with anatomical examination, followed by collection of organs, including the heart, liver, spleen, lungs, kidneys, duodenum, jejunum, ileum, cecum, colon, rectum, skeletal muscle, pancreas, stomach, thymus, testicles, bladder, and eyeballs. The heart, liver, spleen, lungs, kidneys, and testicles were weighed to analyze organic index, whereby organ index = organ weight (g) * 100/body weight (g). The organ tissues were collected with minimum artificial damage in suitable sizes used for further histopathological examination. All obtained rat tissues, in triplicate, from each group were immediately immersed in 4% paraformaldehyde solution, and eyeballs were stored in Davidson’s fixative solution for further histopathological examination as described below.

### Hematological analysis

Hematological changes of hypertensive rats treated with different doses of CQ were detected using an automatic blood counter system (Mindray, Shenzhen, China). These hematological parameters included red blood cell (RBC), hematocrit (HCT), mean red blood cell volume (MCV), hemoglobin (HGB), mean corpuscular hemoglobin (MCH), mean corpuscular hemoglobin concentration (MCHC), RBC distribution width (RDW- SD), white blood cells (WBC), lymphocyte count (LYM), monocytes (MONO), neutrophils (NEUT), eosinophils (EO), basophils (BASO), platelets (PLT) and mean platelet volume (MPV). The obtained plasma samples were analyzed within 4 h of sampling.

### Serum biochemical measurement

We detected serum biochemical changes, including glucose (GLU), total protein (TP), albumin (ALB), globulin (GLB), total bile acid (TBA), alanine aminotransferase (ALT), aspartame acid aminotransferase (AST), total bilirubin (T-BIL), alkaline phosphatase (ALP), lactate dehydrogenase (LDH), creatine kinase (CK), creatinine (Cr), uric acid (UA), total cholesterol (TC), triglycerides (TG), high-density lipoprotein cholesterol (HDL-C), low-density lipoprotein cholesterol (LDL-C), and amylase (AMY). Rat sera, at least in triplicate per group, were tested using an automatic biochemical analyzer (Beckman Coulter AU5800, Brea, CA, United States).

### Urinary assay

Next, routine urine examination was carried out using a urine analyzer (Mindray, Shenzhen, China). The urine parameters include specific gravity, pH, WBC, nitrite, urobilinogen, protein, occult blood, ketone bodies, bilirubin, GLU, and vitamin C were tested and analyzed within 1 h of sample collection. Urine samples, at least in triplicate per group, were analyzed according to the manufacturer’s instructions.

### Serum cytokine assay

To further investigate the effect of CQ on cytokine levels in rat sera, multiple cytokines (TNF-α, IFN-γ, IL-1β, IL-4, IL-6, IL-2, IL-5, GRO/KC, IL-12p70 and IL-10) were analyzed using multiplex immunoassays on a Luminex xMAP (multi-analyte profiling) instrument (Luminex-200, Austin, America) with a commercial kit (LX-MultiDTR-10, LabEx, Shanghai, China) according to the manufacturer’s instructions. In brief, the fluorescently labeled microspheres for different test substances were mixed, and the test substance or the amplified fragments were added to react with the labeled fluorescein. Driven by the flowing sheath fluid, the microspheres were passed through the red and green lasers in sequence in the Luminex-200 system (Luminex, Austin, United States). The obtained fluorescence values were translated to a concentration for the relevant cytokine based on previous established standard curves.

### Histopathological examination

The obtained tissues, in triplicate per group, were used for histopathological analysis. Tissue slice preparation and staining procedures were carried out according to a previous study (Gao et al., 2022). Briefly, the fixed tissues were dehydrated, followed by transparency and embedding in paraffin for cutting into thin slices. The prepared tissue sections were subjected to hematoxylin and eosin (H&E) staining. Histopathological changes of each tissue slice were observed and photographed under a light microscope (Leica, Buffalo Grove, United States).

### Statistical analysis

The data are presented as mean ± standard deviation (SD). Data were analyzed using SPSS (20.0 version, IBM, Chicago, United States) and tested by conducting two-way analysis of variance (ANOVA) along with the least-significant difference (LSD) test. A *p* value less than 0.05 was set as statistical differences in this study. In addition, log-rank (Mantel-Cox) test were performed to analyze the significance between different survival curves.

## Results

### CQ induces toxic effects and sudden death in hypertensive rats

**FIGURE 2:**
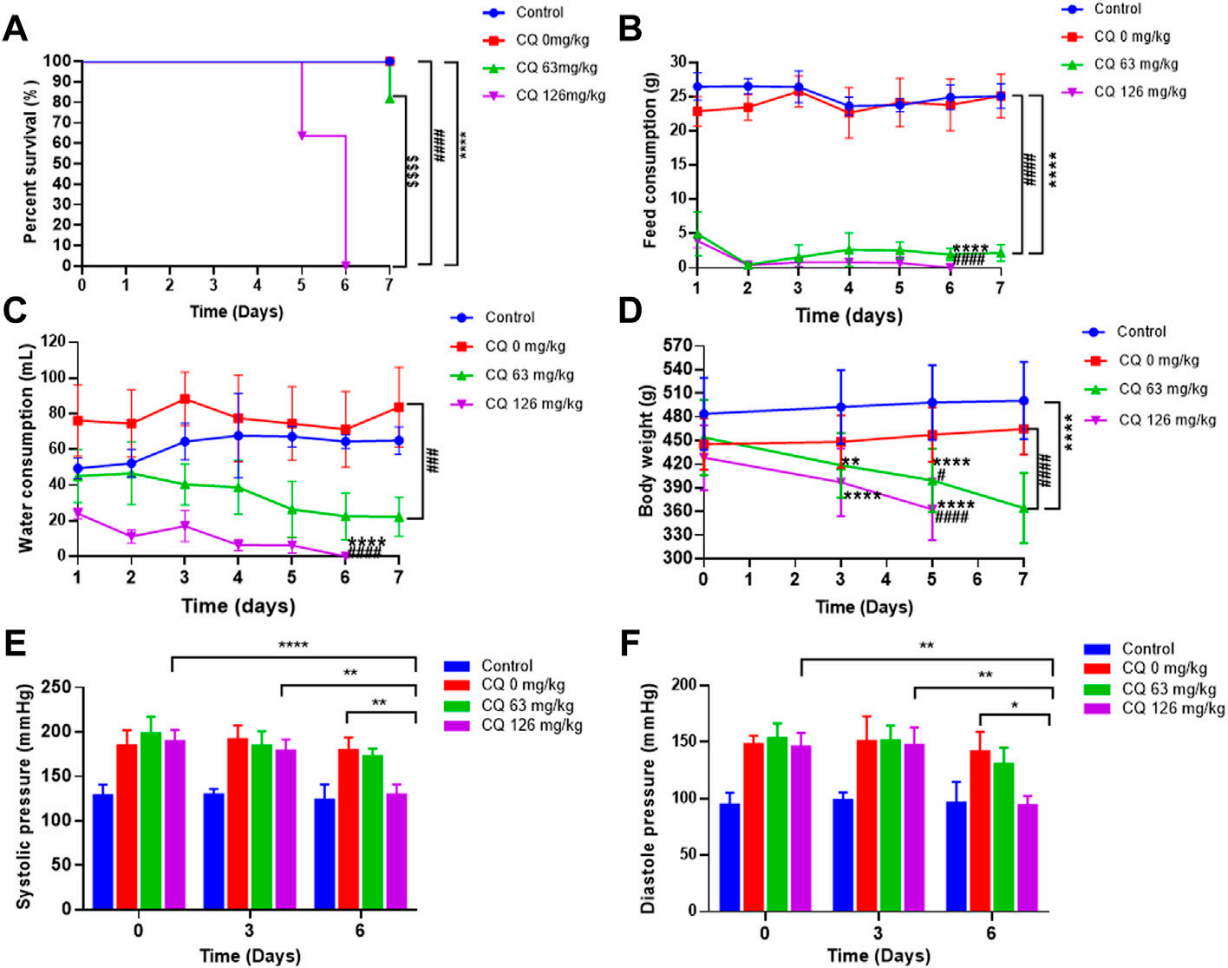
Effect of CQ on survival rate, feed and water consumption, body weight, and blood pressure of rats. Healthy and hypertensive rats orally administrated saline and CQ at two doses (63 mg/kg or 126 mg/kg) twice daily for seven consecutive days. **(A)** The survival rate of rats was recorded daily. ****, *p* < 0.0001, compared to healthy rats; ####, *p* < 0.0001, compared to hypertensive rats; $$$$, *p* < 0.0001, compared to CQ (63 mg/kg) group; Log-rank (Mantel-Cox) test were performed to analyze the significance between different survival curves. **(B, C)** The feed consumption and water consumption of rats were recorded daily. Data are shown as mean ± SD (*n* = 3); ****, *p* < 0.0001, and ####, *p* < 0.0001, compared to healthy and hypertensive rats, respectively;For water consumption, ###, *p* < 0.001, compared with healthy rats. **(D)** The body weight of rats was recorded on the day 0, 3, 5, and 7 of CQ administration. Data are shown as mean ± SD (*n* = 9–12); **, *p* < 0.01, and ****, *p* < 0.0001, compared to healthy rats; #, *p* < 0.05 and ####, *p* < 0.0001, compared to hypertensive rats. **(E, F)** The systolic pressure and diastolic blood pressure of all rats were monitored on 3 d and 6 d after CQ administration. Data are shown as mean ± SD (*n* = 3–12); *, *p* < 0.05; **, *p* < 0.01; ****, *p* < 0.0001.

During the dosing period, clinical signs such as depression, decreased activity, slow response to external stimuli were observed in rats in the 126 mg/kg CQ group on day 3, and the same symptoms were occurred in several rats in the 63 mg/kg CQ group on day 5. With increased CQ exposure, visible weight loss, ruffled fur, prostration, and weakened conditions were noted in most rats treated with both the approved dose and the high dose of CQ. In contrast, there were no abnormal signs in rats in the negative control group and hypertensive rats not receiving CQ. Subsequently, four rats in the high-dose CQ group were found dead on day 5, however, the mortality rate increased dramatically on day 6 after CQ treatment. At the end of CQ administration, compared with the 100% survival rate of hypertensive rats receiving saline, the survival rate of 63 mg/kg and 126 mg/kg CQ treated hypertensive rats decreased to 81.8% and 0% (*p* < 0.0001), respectively (Figure 2A). In addition, the feed intake and water consumption of hypertensive rats declined visibly after CQ treatment (*p* < 0.0001), while the water consumption of hypertensive rats received normal saline increased in comparison with healthy rats (Figures 2B,C). Moreover, the body weight of hypertensive rats decreased dramatically following CQ exposure, especially in the high-dose CQ group (*p* < 0.0001), compared to saline-treated animals (Figure 2D). Furthermore, during the entire administration period, the BP of hypertensive vehicle rats remained high. In contrast, at the last monitoring, the SBP and DBP of hypertensive rats receiving high-dose CQ were significantly decreased (*p* < 0.01), but BP returned to baseline (Figures 2E,F). Finally, as shown in Table 1, CQ increased several organ indexes of hypertensive rats compared to healthy rats and hypertensive rats that received saline, these organs included the heart and liver. Similarly, CQ exposure elevated the kidney index of hypertensive rats (*p* < 0.05), compared to the control group, but not hypertensive rats that received saline. However, other organs such as the spleen, lungs, and testicles of hypertensive rats were not significant changed after CQ treatment.

**TABLE 1:** Organ indexes of hypertensive rats changed after CQ exposure.

Organ	Control	Hypertensive rats		
		CQ (0 mg/kg)	CQ (63 mg/kg)	CQ (126 mg/kg)
Heart	0.303 ± 0.039	0.339 ± 0.031	0.45 ± 0.037^****####^	0.427 ± 0.063^***#^
Liver	2.694 ± 0.203	2.585 ± 0.187	3.134 ± 0.397^#^	3.685 ± 1.052^**##^
Spleen	0.222 ± 0.025	0.196 ± 0.035	0.242 ± 0.074	0.16 ± 0.034
Lung	0.448 ± 0.069	0.461 ± 0.02	0.481 ± 0.068	0.567 ± 0.181
Kidney	0.585 ± 0.066	0.631 ± 0.063	0.787 ± 0.182^*^	0.846 ± 0.17^*^
Testis	0.762 ± 0.097	0.854 ± 0.109	0.914 ± 0.12	0.889 ± 0.119

Note: Data are shown as mean ± SD (*n* = 3–9).

*, *p* < 0.05, compared with control group; ***, *p* < 0.001, compared with control group; ****, *p* < 0.0001, compared with control group; #, *p* < 0.05, compared with CQ (0 mg/kg) group; ##, *p* < 0.01, compared with CQ (0 mg/kg) group; ###, *p* < 0.001, compared with CQ (0 mg/kg) group.

### CQ induces cardiotoxicity of hypertensive rats

**FIGURE 3:**
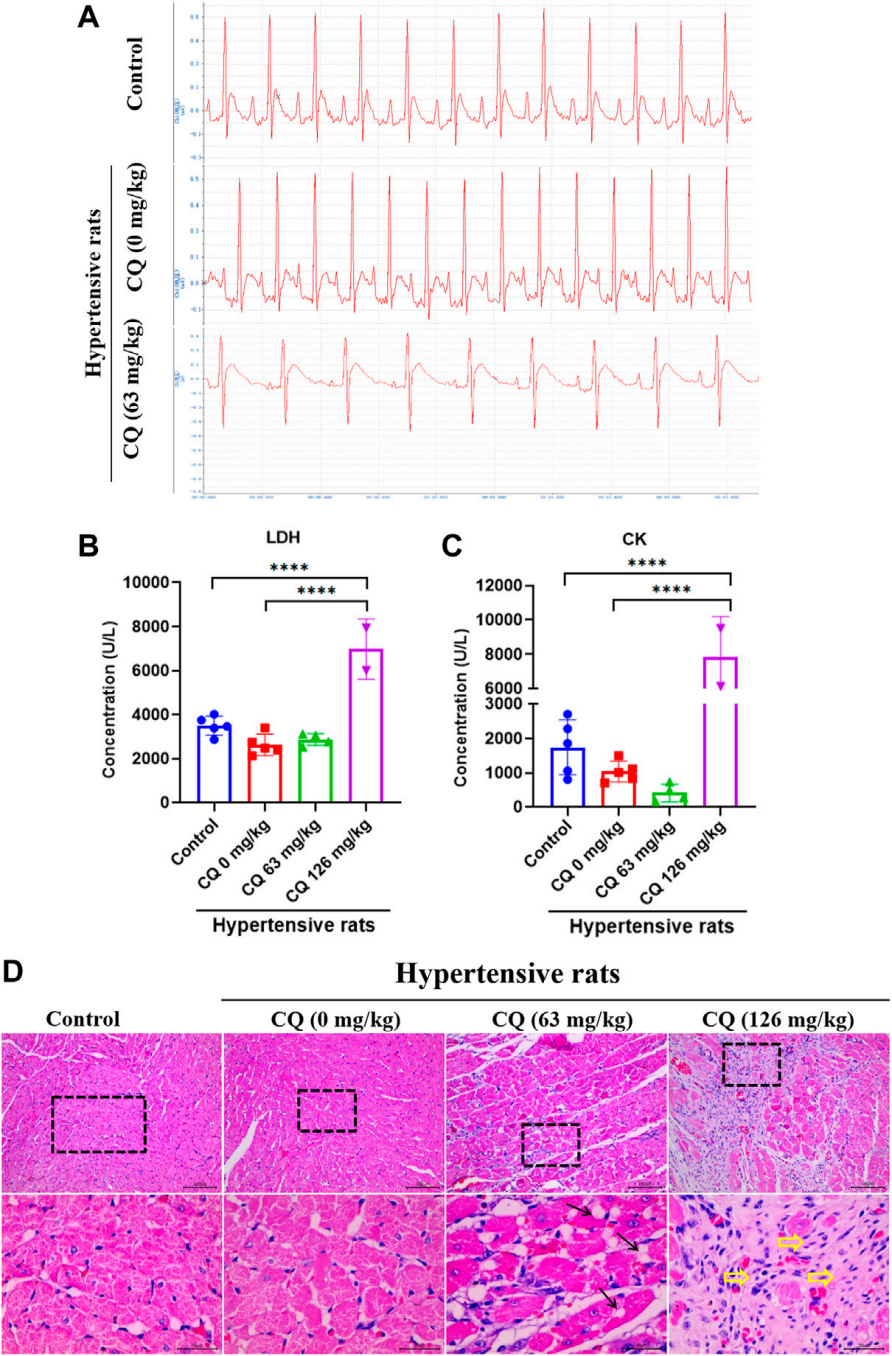
CQ damage to hearts in hypertensive rats. Healthy and hypertensive rats orally administrated saline and CQ at two doses (63 mg/kg or 126 mg/kg) twice daily for seven consecutive days. **(A)** ECG examination of CQ-treated hypertensive rats and rats in control group and negative group. **(B, C)** Effects of CQ on the levels of myocardial enzymes LDH and CK in rats at day 7. Data are shown as mean ± SD (*n* = 3–5); ****, *p* < 0.0001. **(D)** H&E staining of CQ-treated hearts in healthy rats and hypertensive rats at the end of drug administration period; Black arrows indicate vacuolar degeneration and yellow arrowheads present severe myocardial fibrosis and inflammatory cells infiltration; Bars = 30 µm or 100 µm.

Given the high mortality caused by CQ treatment, we next investigated the effect of CQ on the heart in hypertensive rats. Electrocardiogram (ECG) examinations showed that CQ (63 mg/kg) significantly prolonged the QT interval, but not the PR and QRS intervals in hypertensive rats, compared to healthy and hypertensive rats that had received saline (Figure 3A and Table 2). CQ (63 mg/kg) also decreased the heart rate (HR) of hypertensive rats significantly (*p* < 0.05) after the administration period (Table 2). Unfortunately, in this study, we failed to collect ECG parameters of high-dose CQ-treated hypertensive rats due to their poor health conditions and sudden death on day 6. In addition, to further understand the effect of CQ on cardiac functions of rats, we next determined myocardial enzymes and histopathologic changes in CQ-treated hypertensive rats. As shown in Figures 3B,C, high-dose CQ significantly (*p* < 0.0001) elevated LDH and CK levels compared to healthy rats and hypertensive rats that had received saline. However, CQ (63 mg/kg) did not seem to induce any significant changes in LDH and CK in hypertensive rats (Figures 3B,C). Additionally, H&E staining results showed that CQ (63 mg/kg) caused obvious vacuolization degeneration in the hearts of hypertensive rats (black arrows in Figure 3D), and a large area of fibrotic tissue distribution and inflammatory cell infiltration were observed in hypertensive rats treated with CQ (126 mg/kg; yellow arrows in Figure 3D), while the heart slices of healthy rats and hypertensive rats that had received saline showed no marked changes (Figure 3D). In summary, CQ induced severe cardiotoxicity in hypertensive rats, especially at the high dose.

**TABLE 2:** Electrocardiogram changes of hypertensive rats induced by CQ.

Parameter	Control	Hypertensive rats	
		CQ (0 mg/kg)	CQ (63 mg/kg)
PR (ms)	52.17 ± 3.37	52.7 ± 3.75	59.81 ± 6.09
QRS (ms)	24.03 ± 2.77	23.52 ± 5.47	20.42 ± 2.01
QT (ms)	54.77 ± 6.74	66.13 ± 8.77	83.21 ± 12.44^**#^
HR (bpm)	392 ± 38.65	425.2 ± 25.4	330.8 ± 61.02^#^

Note: Data are shown as mean ± SD (*n* = 5); #, *p* < 0.05, compared with CQ (0 mg/kg) group; **, *p* < 0.01, compared with control group.

### CQ induces ocular toxicity in hypertensive rats

**FIGURE 4:**
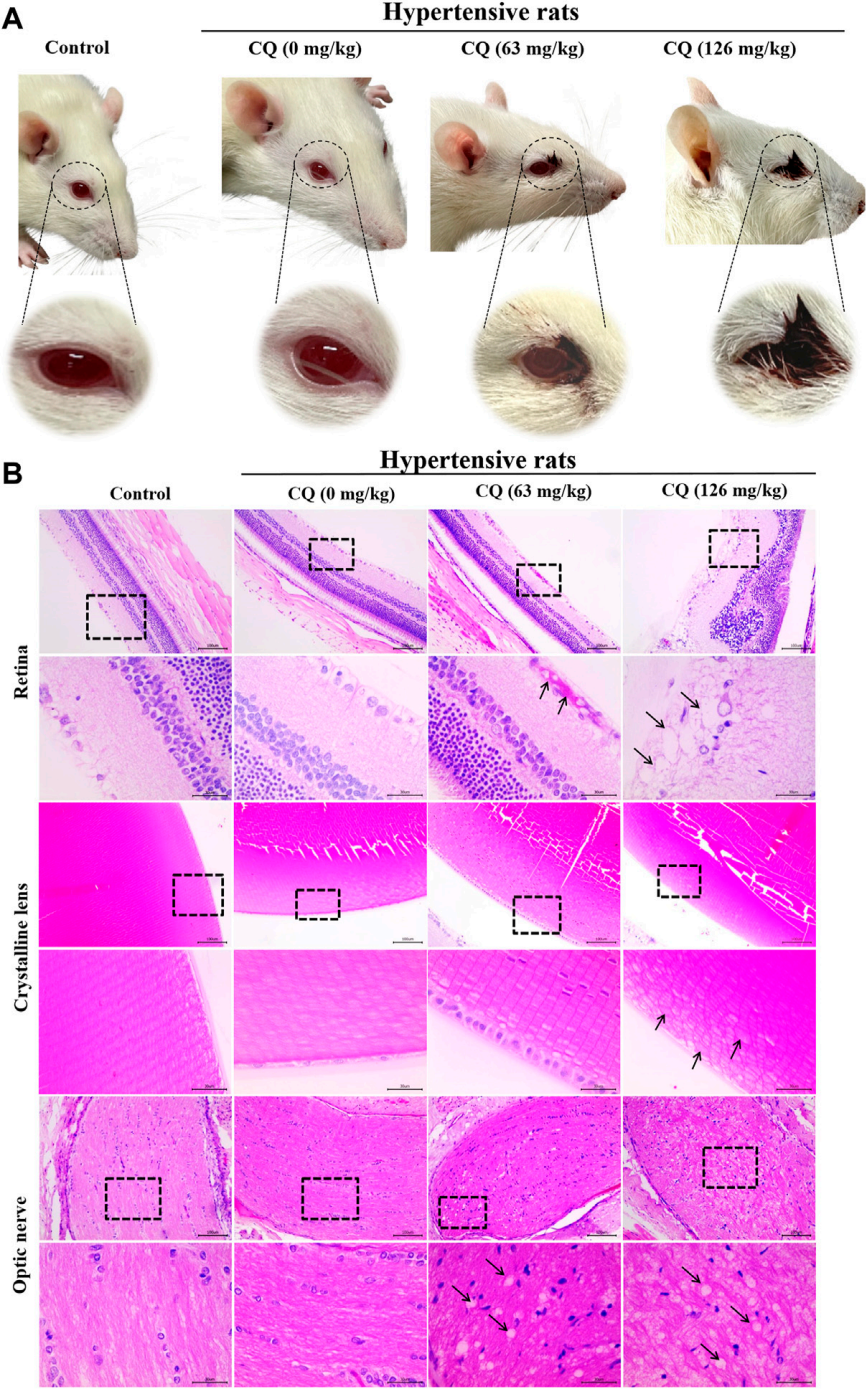
CQ induces ocular toxicity in hypertensive rats. Healthy and hypertensive rats orally administrated saline and CQ at two doses (63 mg/kg or 126 mg/kg) twice daily for seven consecutive days. **(A)** The ocular manifestation in CQ-treated hypertensive rats were observed during the CQ administration period. Blood secretions around eyes and visible hemorrhage on eyeballs were emerged in CQ-treated hypertensive rats. **(B)** Histopathological changes of the components of eyes in CQ-treated hypertensive rats. Rats received high-dose CQ showed obvious vacuolization of retinal nerve fiber layer, and hemorrhage was emerged in the nerve fiber layer of the approved dose CQ group. In high dose CQ group, crystalline lens was in disorder with slight swelling (black arrows). For optic nerve, two doses of CQ resulted in obvious vacuolar degeneration at different degrees in hypertensive rats (black arrows). Bars = 30 µm or 100 µm.

Unfortunately, ocular toxicity was observed during CQ administration period, as shown in Figure 4A, blood secretions appeared in the corner of eyes from day 5 in hypertensive rats. In some severe conditions, blood was visible on the surface of eyeballs in hypertensive rats, especially in the high-dose CQ group on day 6. Subsequent histopathological examination showed that retina, crystalline lens and optic nerves in hypertensive rats were damaged to varying degrees (Figure 4B). Moreover, compared with healthy rats and hypertensive rats, high-dose CQ induced severe retina injuries as evidenced by bleeding in the nerve fiber layer in hypertensive rats treated with the approved dose of CQ, and vacuolar degeneration, as well as abnormal band formation in the inner and outer nuclear layers of high-dose CQ treated hypertensive rats. In addition, compared to animals in the approved CQ dose group, high-dose CQ induced irregular arrangement and slight swelling of the lens in hypertensive rats (Figure 4B). In addition, the optic nerves of hypertensive rats also displayed different degrees of vacuolar degeneration after two doses of CQ treatment. Other components in the eyes of hypertensive rats treated by CQ were not obviously changed (Supplementary Figure S1). In conclusion, CQ induces severe ocular toxicity in hypertensive rats.

### CQ causes gastrointestinal toxicity in hypertensive rats

**FIGURE 5:**
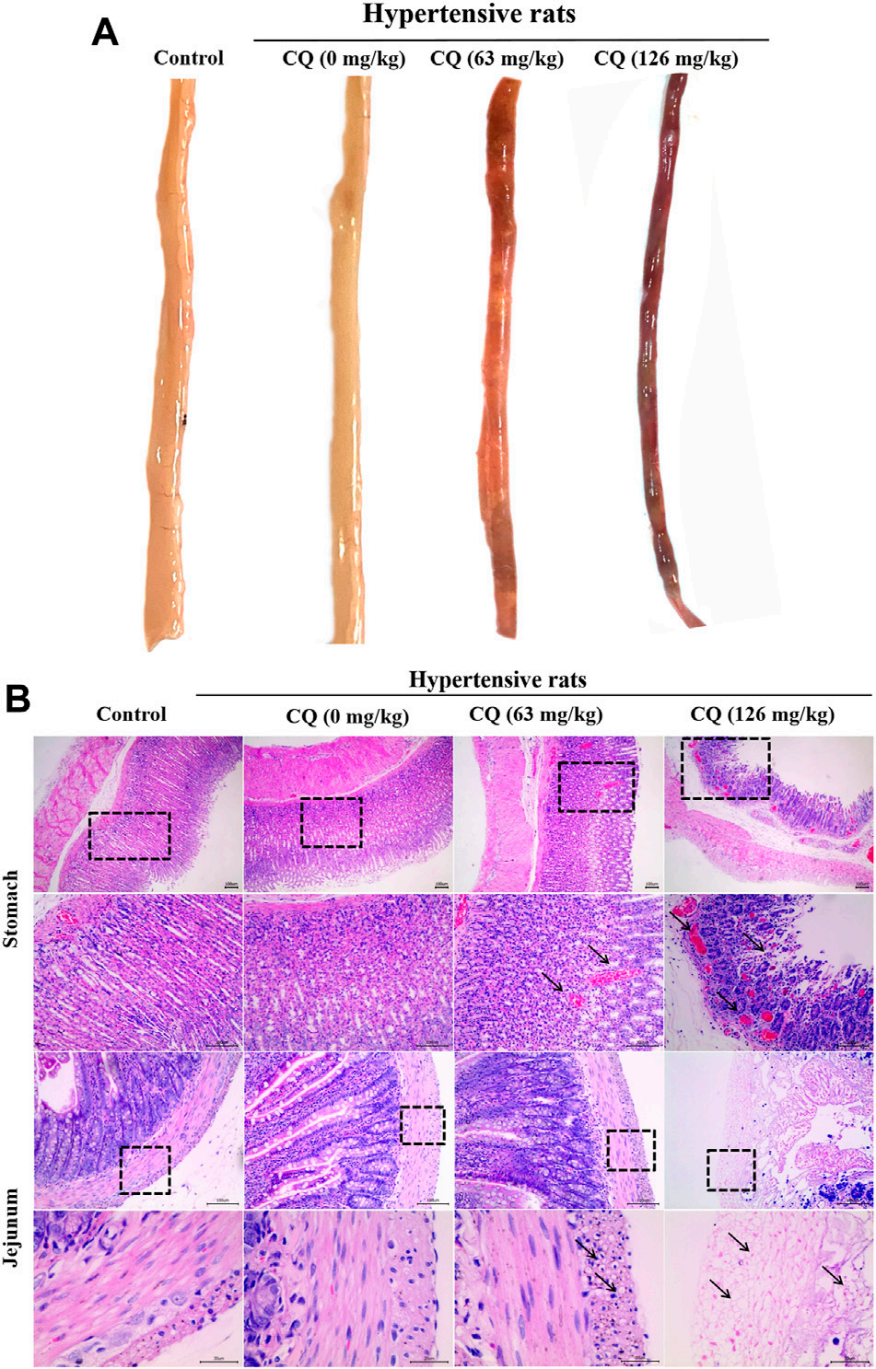
Effect of CQ on gastrointestinal tract of hypertensive rats. Healthy and hypertensive rats orally treated with saline and CQ at two doses (63 mg/kg or 126 mg/kg) twice daily for seven consecutive days. **(A)** Gross autopsy of jejunum in rats treated by CQ. Jejunum became thin and translucent, containing blood related intestinal contents, which was dark red in appearance. **(B)** Histopathological examination of stomach and jejunum in rats treated by CQ. Gastric hemorrhage and gastric villus disruption were found in CQ-treated hypertensive rats (black arrows). Severe vacuolization of intestinal wall and intestinal villus disruption/shedding were observed in CQ-treated hypertensive rats (black arrows). Representative images were obtained from at least triplicate experiments. Bars = 30 µm or 100 µm.

Considering that CQ caused a severe reduction in the feed and water consumption of hypertensive rats, we sought to further investigate whether intestinal toxicity was triggered by CQ. Anatomical examinations of rats revealed jejunums characterized by abnormal states, including thin jejunum walls, which were dark red in appearance with blood-containing intestinal contents (Figure 5A). In contrast, other intestinal segments of CQ-treated hypertensive rats were normal in appearance. In addition, intestinal changes in these rats were further analyzed by histopathological examination on jejunum samples. As shown in Figure 5B, compared to the control and hypertensive group, gastric hemorrhage and gastric villus disruption were observed in the approved- and high-dose CQ-treated hypertensive animals. The approved dose of CQ induced severe vacuolization of the bowel wall and intestinal villus disorder in the jejunums of hypertensive rats. However, high-dose CQ triggered more significant damage, including the shedding of intestinal villus and extreme vacuolization of the bowel wall in hypertensive rats (Figure 5B). Taken together, these data demonstrated that CQ induced varying degrees of gastrointestinal toxicity in hypertensive rats.

### Chloroquine induces hepatotoxicity in hypertensive rats

**FIGURE 6:**
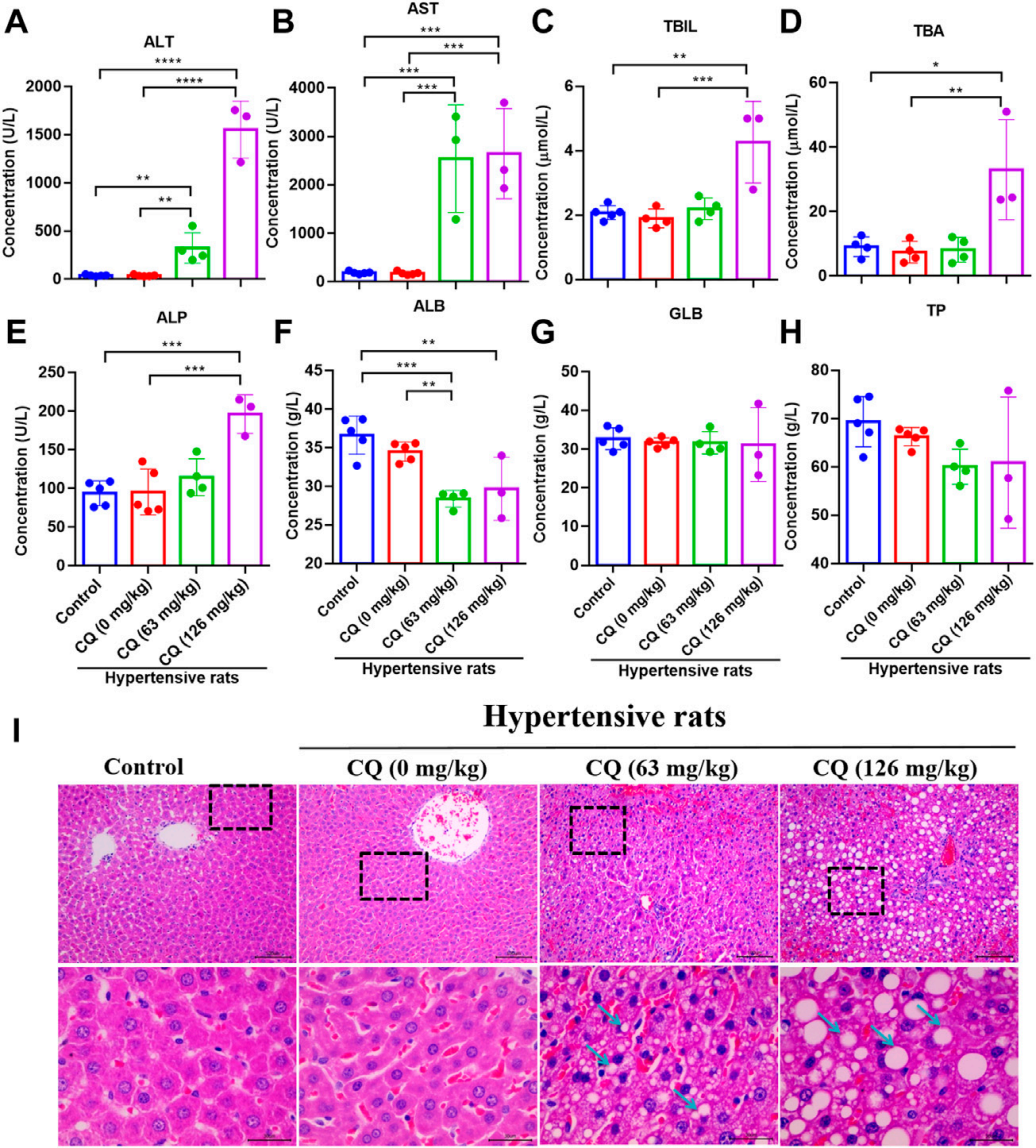
CQ alters hepatic enzymes and damages liver tissues. Healthy and hypertensive rats orally treated with saline and CQ at two doses (63 mg/kg or 126 mg/kg) twice daily for seven consecutive days. **(A-H)** The effects of CQ on hepatic enzymes in serum of hypertensive rats. Data are shown as mean ± SD (*n* = 3–5); ALB, albumin; GLB, globulin; TP, total protein; T-BIL, total bilirubin; AST, aspartate aminotransferase; ALT, alanine aminotransferase; TBA, total bile acid; ALP, alkaline phosphatase. *, *p* < 0.05; **, *p* < 0.01; ***, *p* < 0.001; ****, *p* < 0.0001. **(I)** Histopathological examination of liver in healthy rats and hypertensive rats after CQ treatment. The representative photographs of liver slices, vacuolar degeneration, disorder of hepatic cords and hepatic lobules are indicated by blue arrows. Scale bar was 30 μm or 100 μm.

Next, we evaluated the effect of CQ on the liver in hypertensive rats after the drug administration period. As shown in Figures 6A,B, compared with the negative control and hypertensive rats that had received saline, approved dose CQ significant increased ALT (*p* < 0.01) and AST (*p* < 0.001). Similarly, high-dose CQ also elevated ALT (*p* < 0.0001) and AST (*p* < 0.0001) in hypertensive rats significantly, compared to healthy rats and hypertensive rats that had received saline (Figure 6B). In addition, the levels of T-BIL, TBA and ALP in hypertensive rats were significant increased by high-dose CQ treatment (*p* < 0.05, *p* < 0.01, and *p* < 0.001, respectively), compared to healthy rats and hypertensive rats that had received saline (Figures 6C–E). In contrast, the levels of ALB in hypertensive rats were significant decreased in both CQ treatment groups compared to the negative control and hypertensive rats treated with saline (Figure 6F). However, both doses of CQ did not change the levels of GLB and TP in hypertensive rats (Figures 6G,H). Furthermore, H&E staining showed that the approved dose of CQ induced a disorder of hepatic cords, vacuolar degeneration, which is consistent with hyperchromatic nuclei in liver cells (Figure 6I). The high-dose CQ resulted in more serious vacuolar degeneration of liver cells, along with the disorder of hepatic sinuses and hepatic lobules, and inflammatory infiltration around the central vein (Figure 6I). These above findings suggest that CQ induced dose-dependent liver damage and hepatotoxicity in hypertensive rats.

### Chloroquine triggers nephrotoxicity in hypertensive rats

**FIGURE 7:**
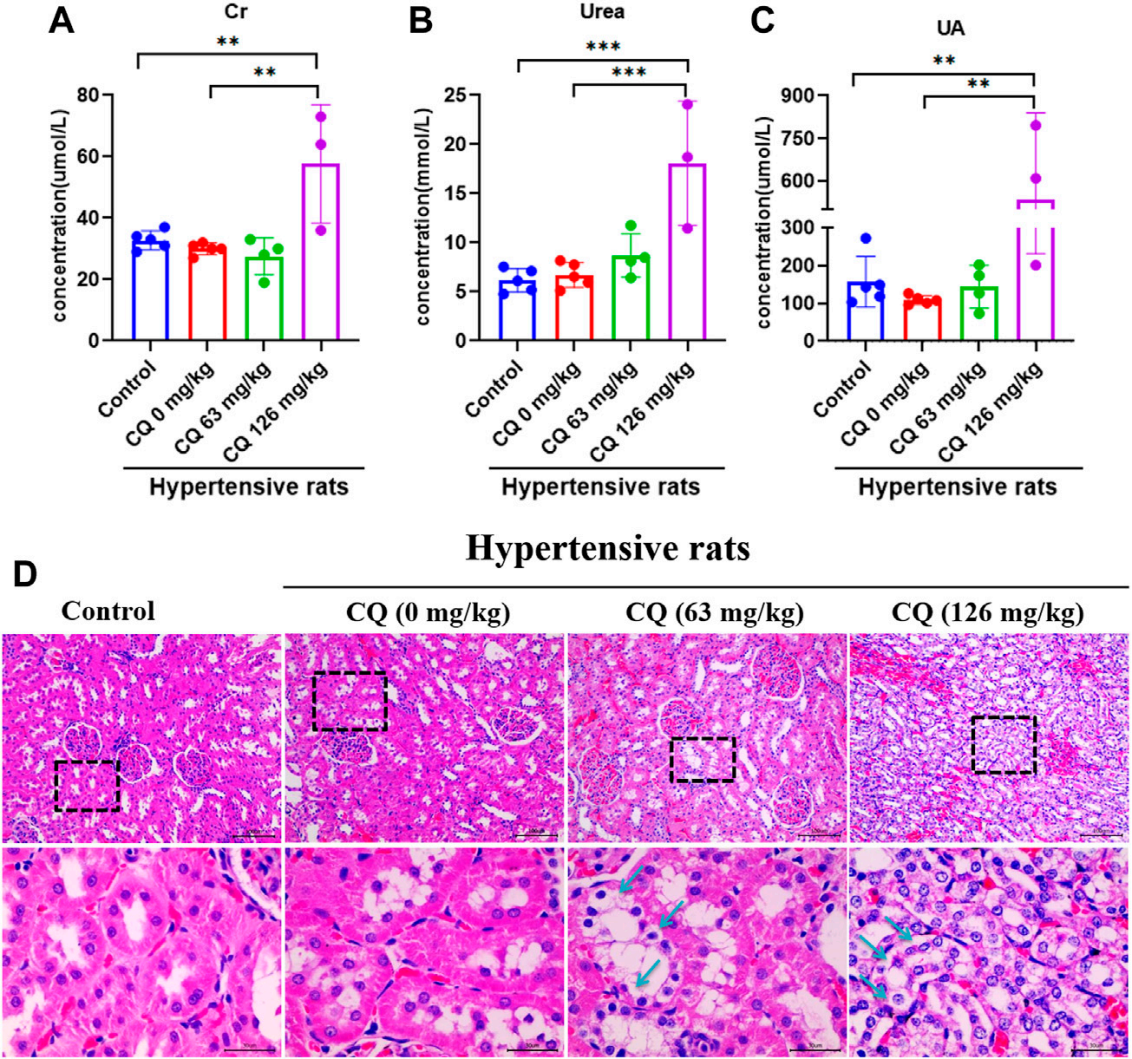
CQ induces nephrotoxicity of hypertensive rats. Healthy and hypertensive rats orally administrated saline and CQ at two doses (63 mg/kg or 126 mg/kg) twice daily for seven consecutive days. **(A–C)** The administration of high-dose CQ (126 mg/kg) elevated the hematological renal function indexes of hypertensive rats. Data are shown as mean ± SD (*n* = 3–5); Cr, creatinine; Ur, urea; UA, uric acid. **, *p* < 0.01; ***, *p* < 0.001. **(D)** CQ induced pathological damage of kidneys in hypertensive rats. Tubular epithelial cells loose connection with hyaline degeneration (black arrows) were observed in kidney slices of hypertensive rats treated with CQ (63 mg/kg), and extensive hyaline degeneration, dilation of kidney tubular lumen, and thinning of renal tubular wall (black arrows) were shown in kidney slices of hypertensive rats exposed to high dose CQ. Bars = 30 µm or 100 µm.

Given the CQ-induced changes in the kidney indices of hypertensive rats, we further investigated the effects of CQ on the kidneys. First, the effect of CQ on urine is shown in Table 3. Compared to healthy and hypertensive rats, leukocyte, protein and occult blood were positive in the urine of both CQ treatment groups. Nevertheless, other urinary parameters such as specific gravity, pH, nitrite, urobilinogen, ketone body, bilirubin, GLU, and vitamin C were not obviously changed after CQ treatment. Moreover, renal functions of hypertensive rats were evaluated by assessing the alterations of serum creatinine (Cr), uric acid (UA), and urea (Ur). As shown in Figures 7A–C, high-dose CQ but not the approved dose enhanced serum Cr, Ur and UA concentrations significantly (*p* < 0.01 or *p* < 0.001), compared to rats in the negative control and hypertensive groups. Finally, histopathological examination of kidneys was carried out and showed that renal tubular epithelial cells lose connection with slight staining following approved dose CQ exposure, and more serious injury of treated renal tubular epithelial cells was observed in high-dose CQ-treated hypertensive rats, showing extensive hyaline degeneration, dilation of kidney tubular lumen, and thinning of the renal tubular wall (Figure 7D). Collectively, these findings show that CQ causes dose-dependent kidney damage.

**TABLE 3:** Effect of CQ on urine indexes in hypertensive rats.

Test items		Control	Hypertensive rats			
			CQ (0 mg/kg)	CQ (63 mg/kg)	CQ (126 mg/kg)
Quantitative measures						
Specific gravity		1.016 ± 0.005	1.023 ± 0.008	1.027 ± 0.004	1.027 ± 0.003	
pH		6.5 ± 0	6.4 ± 0.548	6 ± 0.354	6 ± 0	
Qualitative measures						
Leukocyte	Negative	5	3	1	0	
	Trace	0	1	1	0	
	1+	0	0	3	0	
	2+	0	1	0	2	
	3+	0	0	0	1	
Nitrite	Negative	5	3	5	2	
	1+	0	2	0	1	
Urobilinogen	Negative	5	5	5	3	
Protein	Negative	2	0	0	0	
	Trace	2	0	0	0	
	1+	1	0	0	0	
	2+	0	3	2	0	
	3+	0	2	3	3	
Occult blood	Negative	5	5	2	1	
	1+	0	0	3	0	
	3+	0	0	0	2	
Ketone body	Negative	5	2	4	2	
	Trace	0	3	1	1	
	1+	0	0	0	0	
Bilirubin	Negative	5	5	4	3	
	1+	0	0	1	0	
	2+	0	0	0	0	
Glucose	Negative	5	5	5	3	
Vitamin C	Negative	5	5	5	3	

### Chloroquine alters immune state of hypertensive rats

**FIGURE 8:**
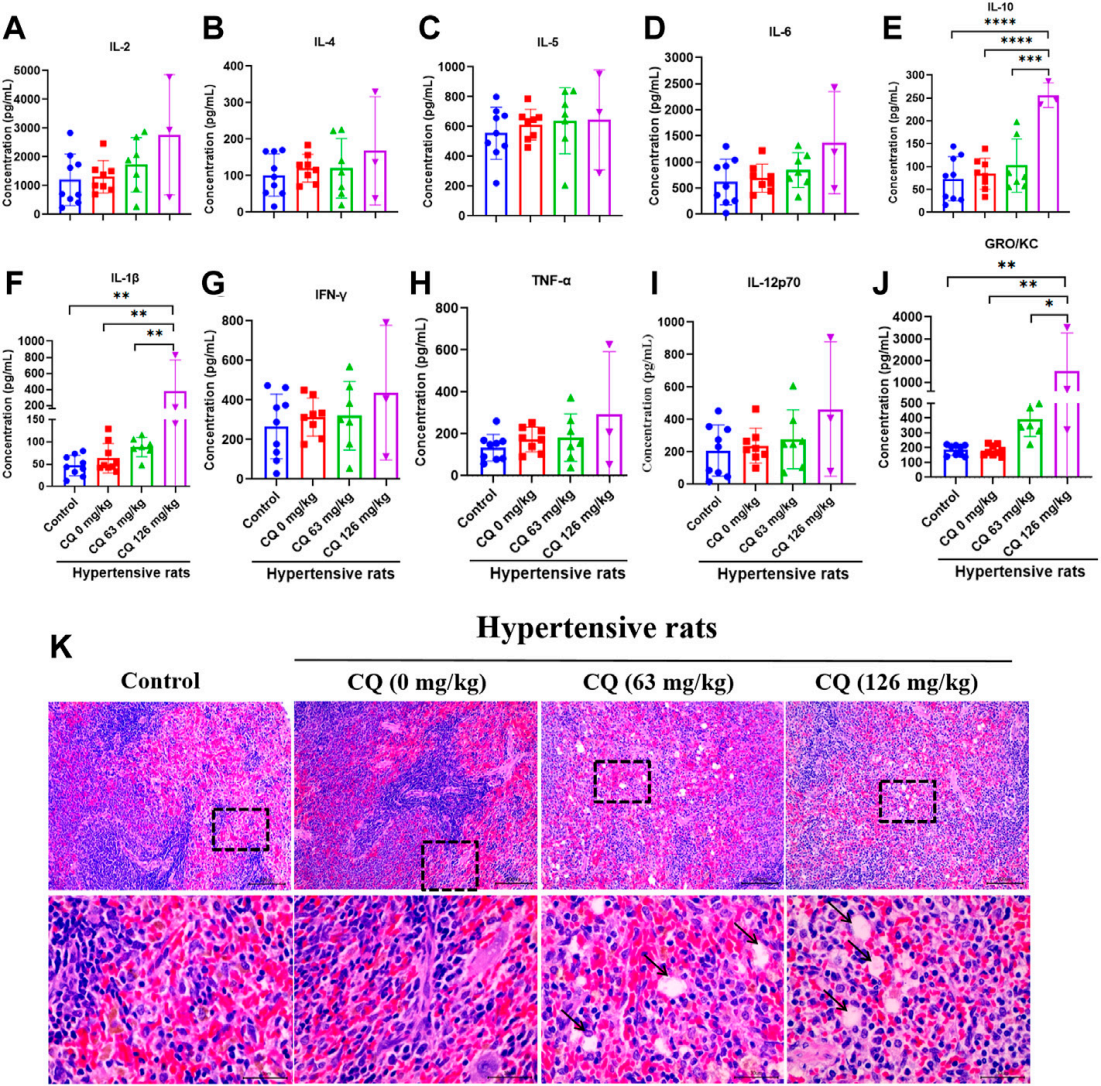
CQ alters immune responses in hypertensive rats. Healthy and hypertensive rats orally treated with saline and CQ at two doses (63 mg/kg or 126 mg/kg) twice daily for seven consecutive days. **(A–J)** After CQ treatment, serum of healthy and hypertensive rats was collected to analyze the levels of several inflammatory cytokines, including IL-2, IL-4, IL-5, IL-6, IL-10, IL-1β, IFN-γ, TNF-α, IL-12p70 and GRO/KC. Data are shown as mean ± SD (*n* = 3–9); *, *p* < 0.05; **, *p* < 0.01; ***, *p* < 0.001; ****, *p* < 0.0001. **(K)** CQ induced spleen pathological damage in hypertensive rats**.** Vacuolar degeneration was observed in hypertensive rats exposed to 63 mg/kg and 126 mg/kg CQ (black arrows). Bars = 30 µm or 100 µm.

Furthermore, we investigated the effect of CQ on immunity in hypertensive rats at the end of the experimental period. First, blood immune cells were counted after CQ treatment (Table 4). Compared to the control and hypertensive groups, CQ significantly (*p* < 0.0001) decreased lymphocyte count in hypertensive rats. However, high-dose CQ treatment significantly increased the levels of monocytes (*p* < 0.0001) and eosinophils (*p* < 0.05 and *p* < 0.0001) in hypertensive rats (Table 4). The approved dose and high-dose CQ both enhanced the levels of neutrophils and basophils significantly, compared to those in the control (*p* < 0.01) and hypertensive groups (*p* < 001 and *p* < 0.001). There were no significant changes in WBCs after CQ treatment (*p* > 0.05). Second, analysis of serum inflammatory cytokines (Figures 8A–J) showed that high-dose CQ but not the approved dose CQ, elevated levels of IL-10 (*p* < 0.001 and *p* < 0.0001), IL-1β (*p* < 0.01) and GRO/KC (*p* < 0.05 and *p* < 0.01) compared to rats in the control and hypertensive groups. Nevertheless, the levels of serum IL-2, IL-4, IL-5, IL-6, IFN-γ, TNF-α, and IL-12p70 in hypertensive rats were not altered after two doses of CQ exposure (Figure 8). Lastly, histopathological changes in the spleens of CQ-treated rats were examined by H&E staining. CQ exposure caused slightly vacuolar degeneration (Figure 8K; black arrows) and slight stained nucleus. These findings suggest that CQ alters immune status and promotes inflammation in hypertensive rats.

**TABLE 4:** Effect of CQ on blood immune cells in hypertensive rats.

Index	Control	Hypertensive rats		
		CQ (0 mg/kg)	CQ (63 mg/kg)	CQ (126 mg/kg)
WBC(10^9/L)	8.59 ± 2.38	8.47 ± 2.07	8.41 ± 3.78	10.84 ± 1.04
LYM(10^9/L)	5.74 ± 1.69	5.35 ± 1.8	1.04 ± 0.99^****,####^	0.76 ± 0.53^****,####^
MONO(10^9/L)	0.27 ± 0.1	0.23 ± 0.1	0.54 ± 0.35	2.19 ± 0.9^****,####,$$$$^
NEUT(10^9/L)	2.27 ± 0.61	2.35 ± 0.53	6.24 ± 2.81^**##^	5.94 ± 1.98^**##^
EO(10^9/L)	0.16 ± 0.08	0.25 ± 0.08	0.05 ± 0.04^*####^	0.27 ± 0.07^*$$$$^
BASO(10^9/L)	0.15 ± 0.05	0.22 ± 0.08	0.73 ± 0.45^**##^	0.81 ± 0.23^***###^

Note: Data are shown as mean ± SD (*n* = 5–8); WBC, white blood cell; LYM, lymphocyte count; MONO, monocyte; NEUT, neutrophils; EO, eosinophils; BASO, basophils.

*p < 0.05, compared with control group; **, *p* < 0.01, compared with control group; ***, *p* < 0.001, compared with control group; ****p < 0.0001, compared with control group.

^##^ *p*<0.01, compared with CQ (0 mg/kg) group.

^###^ *p*<0.001, compared with CQ (0 mg/kg) group.

^####^ *p*<0.0001, compared with CQ (0 mg/kg) group.

^$$$$^ *p*<0.0001, compared with CQ (63 mg/kg) group.

## Discussion

Following the repurposed use of CQ and its derivatives for treating COVID-19, numerous clinical studies and critical reviews have been carried out to evaluate the safety and efficacy of CQ/hydroxychloroquine (Axfors et al., 2021; Das et al., 2021; Junqueira and Rowe, 2021). Although CQ and hydroxychloroquine held some promise as potentially effective anti-SARS-CoV-2 agents during the early stages of the COVID-19 pandemic, they subsequently failed due to safety concerns and a lack of efficacy as shown in international large-scale randomized clinical trials (Consortium et al., 2021). The controversial journey of CQ/hydroxychloroquine prompted a series of scientific questions (Shah, 2021). For example, what is the safety range of CQ in patients with complications? Why did the use of CQ/hydroxychloroquine relate to higher mortality in COVID-19 patients following completion of clinical trials? What toxic events contribute to the failure of CQ in COVID-19 treatment regimens? We sought to answer some of these questions in this work.

Herein, we found that approved- and high-dose CQ induced significant death in hypertensive rats in a concentration-dependent manner. This was consistent with findings obtained from randomized clinical trials of CQ and hydroxychloroquine, showing that a high dosage of CQ (600 mg CQ twice daily for 10 days) caused more toxic effects and lethality in critically ill COVID-19 patients with heart disease (Borba et al., 2020). Unfortunately, our data also agrees with conclusions from the international collaborative meta-analysis in which hydroxychloroquine has been demonstrated to associate with high mortality in COVID-19 patients, while CQ presents no benefits against SARS-CoV-2 infection (Axfors et al., 2021). Although the enrolled patients tolerated low doses of CQ or hydroxychloroquine well in other clinical trials, adverse events are often observed in COVID-19 patients (Self et al., 2020; Rea-Neto et al., 2021; Izcovich et al., 2022). Concerning the narrow safety range of CQ for treating viral diseases, the current approved doses and regimens may contribute to its mortality outcomes in the COVID-19 pandemic (Lacout et al. Perronne, 2021). In this regard, discussions on the dose selection of CQ and hydroxychloroquine were undertaken to provide optimized dose regimens for patients with varied conditions (Cui et al., 2020; Lacout et al., 2021). In addition to the dose regimens of CQ, the comorbid conditions of patients (e.g. heart disease and hypertension) may affect the outcomes of clinical evaluation (Gabor et al., 2021), unfortunately, persons with comorbidities were excluded in most trials, leading to a lack of available data for patients in these subgroups (Skipper et al., 2020; Tang et al., 2020). For hypertensive patients, CQ plays an antihypertension role as shown in one preclinical study (Wu et al., 2017), which is consistent with our data. However, the antihypertension function of CQ was of no benefit in terms of its efficacy in COVID-19 trials. Further studies are required to elucidate the mechanisms behind the antihypertensive effects of CQ.

In addition to the mortality induced by CQ in overdose, several toxic events were also recorded in hypertensive rats. Among these toxicities, cardiotoxicity may play a lethal role because of CQ induced QT interval prolongation related cardiac dysfunction. While CQ-induced cardiac disorder is rare in healthy individuals, this risk increases in patients at high-risk of drug-induced QT prolongation (Tleyjeh et al., 2021). Hypertension is a common complication in COVID-19 patients and may contribute to a high risk of cardiotoxicity, which has been demonstrated in several clinical trials and systematic reviews (Mercuro et al., 2020). Additionally, SARS-CoV-2 infection also induces acute and chronic damage to the cardiovascular system, resulting in a high risk of cardiac dysfunction with concurrent use of QT-prolonging drugs, such as CQ or azithromycin (Zheng et al. Xie, 2020). In this study, we observed sudden death of hypertensive rats following CQ exposure for 5–6 days, especially at high dose, which is closely associated to CQ-induced cardiotoxicity, including QT prolongation, myocardial damage and the release of myocardial enzymes. Taken together, cardiovascular risks must be considered when repurposing CQ and its derivatives for treating newly emerging diseases in persons with comorbidities, such as hypertension (Tleyjeh et al., 2021). In addition, severe ocular toxicity was observed in hypertensive rats treated by the two doses of CQ in this study. According to the recommendation by the American Academy of Ophthalmology, the maximum daily dose of CQ is ≤ 5 mg/kg for HCQ, and ≤2.3 mg/kg for CQ (Marmor et al., 2016). The recommended doses of CQ or HCQ in the guidelines for COVID-19 treatment are considerably higher than the maximum daily safe dose related to ocular toxicity, but this is unlikely to trigger retinal toxicity due to a relatively short exposure time. Considering that CQ is excreted in urine, renal dysfunction acts as a risk factor which increases systemic levels of CQ and the risk of ocular toxicity (Melles and Marmor, 2014). The observed ocular toxicity in hypertensive rats may be the result of CQ-induced renal damage, which we discuss below. Based on the possible ocular toxicity in repurposing CQ, it is our recommendation that risk factors related to retinopathy should be evaluated and excluded to avoid potential retinal toxicity prior to CQ or hydroxychloroquine prescription.

Furthermore, intestinal injury was another severe toxic effect in hypertensive rats caused by the approved dose and high dose of CQ. This adverse effect has been confirmed in clinical trials of CQ in non-ICU hospitalized COVID-19 patients, thereby demonstrating increased gastrointestinal adverse events in CQ-treated patients other than hydroxychloroquine exposure (Verheijen et al., 2021). CQ-induced gastrointestinal toxicity can be detected during early stages of treatment based on the findings that the feed consumption and water intake of hypertensive rats decreased rapidly after 1 day of CQ exposure. During the first wave of the COVID-19 pandemic, reports of CQ- and hydroxychloroquine-associated adverse gastrointestinal events in the FDA Adverse Event Reporting System (FAERS) database increased (Perez et al., 2021), suggesting potential over use of CQ and hydroxychloroquine globally. Self-reported adverse symptoms such as nausea, diarrhea, and abdominal pain were also common effects in healthcare workers during hydroxychloroquine prophylaxis of COVID-19 (Nagaraja et al., 2020). These findings support the hypothesis that treatment with CQ is associated with an increased risk for gastrointestinal toxicities, thus suggesting discontinued use of high-dose CQ for treatment of emerging infectious diseases. However, CQ-induced intestinal toxicity is largely due to high dose treatment and has no relevance to hypertension. Given that the quick absorption of CQ in human body leads to an accumulation of CQ in solid organs such as the liver, heart, and kidneys (White and Watson, 2020), hepatotoxicity and nephrotoxicity are inevitable. In the current study, CQ-induced hepatotoxicity was observed in hypertensive rats including a significant increase of hepatic enzymes and histopathological damage. Hepatotoxicity was reported in a case characterized by a 10-fold increase in aminotransferase and alanine aminotransferase activities after two doses of hydroxychloroquine for treatment of COVID-19 (Falcao, Cavalcanti et al., 2020), but enzyme activities returned to normal after drug discontinuation. However, data related to CQ-induced hepatotoxicity is limited in COVID-19. Considering the use of other drugs such as azithromycin for anti-infection, the potential hepatotoxicity risk of CQ should not be ignored, rather liver function should be monitored during its use. In addition, severe renal injury was only observed in high-dose CQ rather than the approved-dose of CQ-treated hypertensive rats. Prevalence of CQ-induced nephrotoxicity seems to be rare since only a few case reports are available (Mahmoudi et al., 2021). Nevertheless, kidney dysfunction rate in SARS-CoV-2 infected patients is high in hospital (Cheng et al., 2020), which will further enhance the risk of CQ-induced nephrotoxicity. Additionally, direct CQ-mediated kidney damage remains due to its excretion and elimination (Wang et al., 2020), which further results in the accumulation of CQ in the body, leading to other side effects such as retinopathy. Nonetheless, hepatotoxicity and nephrotoxicity are closely related to high dose CQ treatment in the clinic, and are reversible upon drug withdrawal. Concerning the complexity of COVID-19 patient conditions especially in the elderly population with high risk of liver and kidney injury (Gabor et al., 2021), the alternative use of CQ should be approached with extreme caution and only in clinical trials.

In addition to the above discussed toxic effects, the immune-regulation function of CQ in hypertensive rats was observed in this study. We found that high-dose rather than the approved dose of CQ increased serum IL-10, IL-1β, and GRO. Since CQ and its derivatives are widely used for treating autoimmune diseases, the anti-inflammatory and immunomodulatory effects of CQ are beneficial for chronic inflammatory and autoimmune diseases over long term use (Rainsford et al., 2015). However, high-dose CQ significantly elevated some inflammatory cytokines, thereby presenting a potential risk of immune damage in hypertensive rats. Moreover, inflammatory markers such as IL-10 and GRO increased significantly in hospitalized COVID-19 patients (Savarraj et al., 2021), which will result in worse situations if these patients were treated with a high dose of CQ. This harmful effect of CQ on the immune system has been linked to endothelial cell injury, and may contribute to its failure in clinical trials during the COVID-19 pandemic (Gregorio et al., 2021). Therefore, the effects of CQ and its derivatives on the immune system especially in SARS-CoV-2 infected patients merit further investigation by analyzing data obtained from largely randomized clinical trials. The undesirable effects of CQ, particularly at high dose, have been demonstrated in hypertensive rats, and partly address its lack of clinical efficacy against COVID-19. Our study still has several limitations: 1) hypertensive rats were not infected by SARS-CoV-2 meaning we could not absolutely model COVID-19 patients complicated with hypertension; 2) the derivate of CQ with low toxicity, hydroxychloroquine, has not been evaluated in the current study; 3) the appropriate safe dose of CQ for hypertensive patients was not proposed based on the regimen used in this study. Despite these limitations, our findings aid our understanding of the failure reasons of CQ against COVID-19 due to its safety concerns, and emphasize that safety evaluation of CQ is a necessary step before its off-label use in the treatment of emerging infectious diseases.

## Conclusion

In conclusion, the current dose and regimen of CQ used in trials for COVID-19 is not safe in hypertensive rats. The approved dose and high dose of CQ both increase the risk of mortality in hypertensive rats. CQ induces multiple toxic effects in hypertensive rats, among which, cardiotoxicity, ocular toxicity and intestinal toxicity are the most severe adverse effects. Furthermore, hepatotoxicity, nephrotoxicity and immune state alteration are related to overdose exposure of CQ in hypertensive rats. These findings collectively explain the failure reason of CQ in the battle against SARS-CoV-2, and underline the importance of safety evaluation and medical supervision of CQ to limit patient harm especially those with hypertension.

## Data Availability

The original contributions presented in the study are included in the article/Supplementary Material, further inquiries can be directed to the corresponding author.

## References

[B1] Adaramoye O. A. Nwosu I. O. Farombi E. O. (2012). Sub-acute effect of N(G)-nitro-l-arginine methyl-ester (L-NAME) on biochemical indices in rats: Protective effects of Kolaviron and extract of Curcuma longa L. Pharmacogn. Res. 4 (3), 127–133. 10.4103/0974-8490.99071 PMC3424838 22923949

[B4] Axfors C. Schmitt A. M. Janiaud P. Van't Hooft J. Abd-Elsalam S. Abdo E. F. (2021). Mortality outcomes with hydroxychloroquine and chloroquine in COVID-19 from an international collaborative meta-analysis of randomized trials. Nat. Commun. 12 (1), 2349. 10.1038/s41467-021-22446-z 33859192 PMC8050319

[B5] Bader M. (2010). Rat models of cardiovascular diseases. Methods Mol. Biol. 597, 403–414. 10.1007/978-1-60327-389-3_27 20013248

[B6] Ball D. E. Tagwireyi D. Nhachi C. F. (2002). Chloroquine poisoning in Zimbabwe: A toxicoepidemiological study. J. Appl. Toxicol. 22 (5), 311–315. 10.1002/jat.864 12355560

[B7] Borba M. G. S. Val F. F. A. Sampaio V. S. Alexandre M. A. A. Melo G. C. Brito M. (2020). Effect of high vs low doses of chloroquine diphosphate as adjunctive therapy for patients hospitalized with severe acute respiratory syndrome coronavirus 2 (SARS-CoV-2) infection: A randomized clinical trial. JAMA Netw. Open 3 (4), e208857. 10.1001/jamanetworkopen.2020.8857 32330277 PMC12124691

[B8] Boulware D. R. Pullen M. F. Bangdiwala A. S. Pastick K. A. Lofgren S. M. Okafor E. C. (2020). A randomized trial of hydroxychloroquine as postexposure prophylaxis for covid-19. N. Engl. J. Med. 383 (6), 517–525. 10.1056/NEJMoa2016638 32492293 PMC7289276

[B9] Cheng Y. Luo R. Wang K. Zhang M. Wang Z. Dong L. (2020). Kidney disease is associated with in-hospital death of patients with COVID-19. Kidney Int. 97 (5), 829–838. 10.1016/j.kint.2020.03.005 32247631 PMC7110296

[B12] Clemessy J. L. Taboulet P. Hoffman J. R. Hantson P. Barriot P. Bismuth C. (1996). Treatment of acute chloroquine poisoning: A 5-year experience. Crit. Care Med. 24 (7), 1189–1195. 10.1097/00003246-199607000-00021 8674334

[B13] Consortium W. H. O. S. T. Pan H. Peto R. Henao-Restrepo A. M. Preziosi M. P. Sathiyamoorthy V. (2021). Repurposed antiviral drugs for covid-19 - interim WHO solidarity trial results. N. Engl. J. Med. 384 (6), 497–511. 10.1056/NEJMoa2023184 33264556 PMC7727327

[B14] Cui C. Zhang M. Yao X. Tu S. Hou Z. Jie En V. S. (2020). Dose selection of chloroquine phosphate for treatment of COVID-19 based on a physiologically based pharmacokinetic model. Acta Pharm. Sin. B 10 (7), 1216–1227. 10.1016/j.apsb.2020.04.007 32834950 PMC7252145

[B15] Das S. Ramachandran A. K. Birangal S. R. Akbar S. Ahmed B. Joseph A. (2021). The controversial therapeutic journey of chloroquine and hydroxychloroquine in the battle against SARS-CoV-2: A comprehensive review. Med. Drug Discov. 10, 100085. 10.1016/j.medidd.2021.100085 33846702 PMC8026171

[B16] Dinesh B. J C. S. Kaur C. P. Atiya F. Yk G. Avijit H. (2021). Hydroxychloroquine for sars CoV2 prophylaxis in healthcare workers - a multicentric cohort study assessing effectiveness and safety. J. Assoc. Physicians India 69 (6), 11–12. Available at: http://www.ncbi.nlm.nih.gov/pubmed/34472778 . 34472778

[B3] European Medicines Agency (2020). COVID-19: Chloroquine and hydroxychloroquine only to Be used in clinical trials or emergency use programs. Available at: https://www.ema.europa.eu/en/documents/press-release/covid-19-chloroquinehydroxychloroquine-only-be-used-clinical-trials-emergency-use-programmes_en.pdf .

[B17] Falcao M. B. Cavalcanti L. P. D. Filgueiras N. M. de Brito C. A. A. (2020). Case report: Hepatotoxicity associated with the use of hydroxychloroquine in a patient with COVID-19. Am. J. Trop. Med. Hyg. 102 (6), 1214–1216. 10.4269/ajtmh.20-0276 32314698 PMC7253107

[B18] Gabor J. J. Kreidenweiss A. Weber S. Salama M. Sulyok M. Sulyok Z. (2021). A call to caution when hydroxychloroquine is given to elderly patients with COVID-19. Int. J. Infect. Dis. 106, 265–268. 10.1016/j.ijid.2021.04.009 33848675 PMC8035801

[B19] Gao X. Jing X. Wang J. Zheng Y. Qiu Y. Ji H. (2022). Safety considerations of chloroquine in the treatment of patients with diabetes and COVID-19. Chem. Biol. Interact. 361, 109954. 10.1016/j.cbi.2022.109954 35469826 PMC9023373

[B20] Grasselli G. Greco M. Zanella A. Albano G. Antonelli M. Bellani G. (2020). Risk factors associated with mortality among patients with COVID-19 in intensive care units in lombardy, Italy. JAMA Intern. Med. 180 (10), 1345–1355. 10.1001/jamainternmed.2020.3539 32667669 PMC7364371

[B21] Gregorio P. da Cunha R. S. Biagini G. Bosquetti B. Budag J. Ortiz A. (2021). Chloroquine may induce endothelial injury through lysosomal dysfunction and oxidative stress. Toxicol. Appl. Pharmacol. 414, 115412. Artn 115412. 10.1016/j.taap.2021.115412 33484708 PMC7826090

[B22] Hoffmann M. Mosbauer K. Hofmann-Winkler H. Kaul A. Kleine-Weber H. Kruger N. (2020). Chloroquine does not inhibit infection of human lung cells with SARS-CoV-2. Nature 585 (7826), 588–590. 10.1038/s41586-020-2575-3 32698190

[B23] Huang M. Li M. Xiao F. Pang P. Liang J. Tang T. (2020). Preliminary evidence from a multicenter prospective observational study of the safety and efficacy of chloroquine for the treatment of COVID-19. Natl. Sci. Rev. 7 (9), 1428–1436. 10.1093/nsr/nwaa113 34676087 PMC7313782

[B24] Izcovich A. Siemieniuk R. A. Bartoszko J. J. Ge L. Zeraatkar D. Kum E. (2022). Drug treatments for Covid-19: Living systematic review and network meta-analysis. BMJ Open 12 (3), m2980. 10.1136/bmj.m2980 PMC7390912 32732190

[B25] Junqueira D. R. Rowe B. H. (2021). Efficacy and safety outcomes of proposed randomized controlled trials investigating hydroxychloroquine and chloroquine during the early stages of the COVID-19 pandemic. Br. J. Clin. Pharmacol. 87 (4), 1758–1767. 10.1111/bcp.14598 33047848 PMC7675266

[B26] Lacout A. Lounnas V. Perronne C. (2021). Timing and dosage may be the key in the realisation of hydroxychloroquine + azithromycin treatment benefit in Covid-19 elderly patients. Int. J. Antimicrob. Agents 57 (4), 106314. ARTN 106314. 10.1016/j.ijantimicag.2021.106314 33722657 PMC7967398

[B27] Lerman L. O. Kurtz T. W. Touyz R. M. Ellison D. H. Chade A. R. Crowley S. D. (2019). Animal models of hypertension: A scientific statement from the American heart association. Hypertension 73 (6), e87–e120. 10.1161/hyp.0000000000000090 30866654 PMC6740245

[B28] Mahmoudi J. Sadigh-Eteghad S. Salehi-Pourmehr H. Gharekhani A. Ziaee M. (2021). Nephrotoxicity of chloroquine and hydroxychloroquine in COVID-19 patients. Adv. Pharm. Bull. 11 (1), 6–7. 10.34172/apb.2021.022 33747847 PMC7961219

[B29] Marmor M. F. Kellner U. Lai T. Y. Melles R. B. Mieler W. F. American Academy of O. (2016). Recommendations on screening for chloroquine and hydroxychloroquine retinopathy (2016 revision). Ophthalmology 123 (6), 1386–1394. 10.1016/j.ophtha.2016.01.058 26992838

[B30] Megarbane B. (2021). Chloroquine and hydroxychloroquine to treat COVID-19: Between hope and caution. Clin. Toxicol. 59 (1), 70–71. 10.1080/15563650.2020.1748194 32237918

[B31] Melles R. B. Marmor M. F. (2014). The risk of toxic retinopathy in patients on long-term hydroxychloroquine therapy. JAMA Ophthalmol. 132 (12), 1453–1460. 10.1001/jamaophthalmol.2014.3459 25275721

[B32] Mercuro N. J. Yen C. F. Shim D. J. Maher T. R. McCoy C. M. Zimetbaum P. J. (2020). Risk of QT interval prolongation associated with use of hydroxychloroquine with or without concomitant azithromycin among hospitalized patients testing positive for coronavirus disease 2019 (COVID-19). JAMA Cardiol. 5 (9), 1036–1041. 10.1001/jamacardio.2020.1834 32936252 PMC7195692

[B33] Multicenter collaboration group of Department of Science and Technology of Guangdong Province and Health Commission of Guangdong Province for chloroquine in the treatment of novel coronavirus pneumonia (2020). Expert consensus on chloroquine phosphate for the treatment of novel coronavirus pneumonia. Zhonghua Jie He He Hu Xi Za Zhi 43 (0), E019. 10.3760/cma.j.issn.1001-0939.2020.0019 32075365

[B34] Nagaraja B. S. Ramesh K. N. Dhar D. Mondal M. S. Dey T. Saha S. (2020). HyPE study: Hydroxychloroquine prophylaxis-related adverse events' analysis among healthcare workers during COVID-19 pandemic: A rising public health concern. J. Public Health 42 (3), 493–503. 10.1093/pubmed/fdaa074 PMC7313915 32490532

[B10] National Health Commission of the People’s Republic of China (2020). Diagnosis and treatment of COVID-19 in China (the six edition for trial). Available at: http://www.nhc.gov.cn/yzygj/s7653p/202002/8334a8326dd94d329df351d7da8aefc2.shtml . 10.46234/ccdcw2020.082 PMC8392946 34594648

[B11] National Health Commission of the People’s Republic of China (2021). Diagnosis and treatment Program of COVID-19 in China(the ninth edition for trial). Available at: http://www.nhc.gov.cn/xcs/zhengcwj/202003/46c9294a7dfe4cef80dc7f5912eb1989.shtml .

[B35] Nirk E. L. Reggiori F. Mauthe M. (2020). Hydroxychloroquine in rheumatic autoimmune disorders and beyond. EMBO Mol. Med. 12 (8), e12476. 10.15252/emmm.202012476 32715647 PMC7411564

[B36] Organization W. H. (2015). Guidelines for the treatment of malaria. 3rd edition. Geneva.

[B37] Peng M. He J. Xue Y. Yang X. Liu S. Gong Z. (2021). Role of hypertension on the severity of COVID-19: A review. J. Cardiovasc. Pharmacol. 78 (5), e648–e655. 10.1097/FJC.0000000000001116 34321401 PMC8562915

[B38] Perez J. Roustit M. Lepelley M. Revol B. Cracowski J. L. Khouri C. (2021). Reported adverse drug reactions associated with the use of hydroxychloroquine and chloroquine during the COVID-19 pandemic. Ann. Intern. Med. 174 (6), 878–880. 10.7326/M20-7918 33493009 PMC7847720

[B39] Rainsford K. D. Parke A. L. Clifford-Rashotte M. Kean W. F. (2015). Therapy and pharmacological properties of hydroxychloroquine and chloroquine in treatment of systemic lupus erythematosus, rheumatoid arthritis and related diseases. Inflammopharmacology 23 (5), 231–269. 10.1007/s10787-015-0239-y 26246395

[B40] Rea-Neto A. Bernardelli R. S. Camara B. M. D. Reese F. B. Queiroga M. V. O. Oliveira M. C. (2021). An open-label randomized controlled trial evaluating the efficacy of chloroquine/hydroxychloroquine in severe COVID-19 patients. Sci. Rep. 11 (1), 9023. 10.1038/s41598-021-88509-9 33907251 PMC8079411

[B41] Rees D. D. Palmer R. M. Schulz R. Hodson H. F. Moncada S. (1990). Characterization of three inhibitors of endothelial nitric oxide synthase *in vitro* and *in vivo* . Br. J. Pharmacol. 101 (3), 746–752. 10.1111/j.1476-5381.1990.tb14151.x 1706208 PMC1917753

[B42] Reeves P. G. Nielsen F. H. Fahey G. C. (1993). Ain-93 purified diets for laboratory rodents - final report of the American institute of nutrition ad hoc writing committee on the reformulation of the ain-76a rodent diet. J. Nutr. 123 (11), 1939–1951. 10.1093/jn/123.11.1939 8229312

[B43] Riou B. Barriot P. Rimailho A. Baud F. J. (1988). Treatment of severe chloroquine poisoning. N. Engl. J. Med. 318 (1), 1–6. 10.1056/NEJM198801073180101 3336379

[B44] Rosenberg E. S. Dufort E. M. Udo T. Wilberschied L. A. Kumar J. Tesoriero J. (2020). Association of treatment with hydroxychloroquine or azithromycin with in-hospital mortality in patients with COVID-19 in New York state. JAMA 323 (24), 2493–2502. 10.1001/jama.2020.8630 32392282 PMC7215635

[B45] Savarraj J. Park E. S. Colpo G. D. Hinds S. N. Morales D. Ahnstedt H. (2021). Brain injury, endothelial injury and inflammatory markers are elevated and express sex-specific alterations after COVID-19. J. Neuroinflammation 18 (1), 277. 10.1186/s12974-021-02323-8 34838058 PMC8627162

[B46] Self W. H. Semler M. W. Leither L. M. Casey J. D. Angus D. C. Brower R. G. (2020). Effect of hydroxychloroquine on clinical status at 14 Days in hospitalized patients with COVID-19: A randomized clinical trial. JAMA 324 (21), 2165–2176. 10.1001/jama.2020.22240 33165621 PMC7653542

[B47] Shah R. R. (2021). Chloroquine and hydroxychloroquine for COVID-19: Perspectives on their failure in repurposing. J. Clin. Pharm. Ther. 46 (1), 17–27. 10.1111/jcpt.13267 32981089 PMC7537228

[B48] Skipper C. P. Pastick K. A. Engen N. W. Bangdiwala A. S. Abassi M. Lofgren S. M. (2020). Hydroxychloroquine in nonhospitalized adults with early COVID-19 : A randomized trial. Ann. Intern. Med. 173 (8), 623–631. 10.7326/M20-4207 32673060 PMC7384270

[B49] Slater A. F. (1993). Chloroquine: Mechanism of drug action and resistance in Plasmodium falciparum. Pharmacol. Ther. 57 (2-3), 203–235. 10.1016/0163-7258(93)90056-j 8361993

[B50] Tang W. Cao Z. J. Han M. F. Wang Z. Y. Chen J. W. Sun W. J. (2020). Hydroxychloroquine in patients with mainly mild to moderate coronavirus disease 2019: Open label, randomised controlled trial. Bmj-British Med. J. 369, m1849. Artn M1849. 10.1136/bmj.m1849 PMC7221473 32409561

[B51] Tleyjeh I. M. Kashour Z. AlDosary O. Riaz M. Tlayjeh H. Garbati M. A. (2021). Cardiac toxicity of chloroquine or hydroxychloroquine in patients with COVID-19: A systematic review and meta-regression analysis. Mayo Clin. Proc. Innov. Qual. Outcomes 5 (1), 137–150. 10.1016/j.mayocpiqo.2020.10.005 33163895 PMC7605861

[B2] U.S. Food & Drug Administration (2020). Request for emergency use authorization for use of chloroquine phosphate or hydroxychloroquine sulfate supplied from the strategic national stockpile for treatment of 2019 coronavirus disease. Available at: https://www.fda.gov/media/136534 .

[B52] Verheijen S. van Luin M. Bruggemann R. J. de Mast Q. Hassing R. J. Burger D. M. (2021). More gastro-intestinal adverse events in non-ICU hospitalised COVID-19 patients treated with chloroquine versus hydroxychloroquine. Int. J. Infect. Dis. 103, 402–403. 10.1016/j.ijid.2020.12.010 33310106 PMC7836616

[B53] Wang B. Guo H. Ling L. Ji J. Niu J. Gu Y. (2020). The chronic adverse effect of chloroquine on kidney in rats through an autophagy dependent and independent pathways. Nephron 144 (2), 96–108. 10.1159/000503882 31661702

[B54] White N. J. Watson J. A. Hoglund R. M. Chan X. H. S. Cheah P. Y. Tarning J. (2020). COVID-19 prevention and treatment: A critical analysis of chloroquine and hydroxychloroquine clinical pharmacology. PLoS Med. 17 (9), e1003252. 10.1371/journal.pmed.1003252 32881895 PMC7470382

[B55] Wiersinga W. J. Rhodes A. Cheng A. C. Peacock S. J. Prescott H. C. (2020). Pathophysiology, transmission, Diagnosis, and treatment of coronavirus disease 2019 (COVID-19): A review. JAMA 324 (8), 782–793. 10.1001/jama.2020.12839 32648899

[B56] Wu K. Zhang Q. Wu X. T. Lu W. J. Tang H. Y. Liang Z. H. (2017). Chloroquine is a potent pulmonary vasodilator that attenuates hypoxia-induced pulmonary hypertension. Br. J. Pharmacol. 174 (22), 4155–4172. 10.1111/bph.13990 28849593 PMC5659991

[B57] Xu S. Xu S. S. Bian R. Chen X. Rulian K. Xu S. Y. (2002). Experimental methodology of Pharmacology. Beijing: People’s Health Publisher.

[B58] Zheng Y. Y. Ma Y. T. Zhang J. Y. Xie X. (2020). COVID-19 and the cardiovascular system. Nat. Rev. Cardiol. 17 (5), 259–260. 10.1038/s41569-020-0360-5 32139904 PMC7095524

[B59] Zhou F. Yu T. Du R. Fan G. Liu Y. Liu Z. (2020). Clinical course and risk factors for mortality of adult inpatients with COVID-19 in wuhan, China: A retrospective cohort study. Lancet 395 (10229), 1054–1062. 10.1016/S0140-6736(20)30566-3 32171076 PMC7270627

