# Supplementary figures and images for: Hypertension related toxicity of chloroquine explains its failure against COVID-19: Based on rat model
Source: Front Pharmacol. 2022 Nov 30;13:1051694. doi: 10.3389/fphar.2022.1051694 (PMC9748293; doi:10.3389/fphar.2022.1051694)

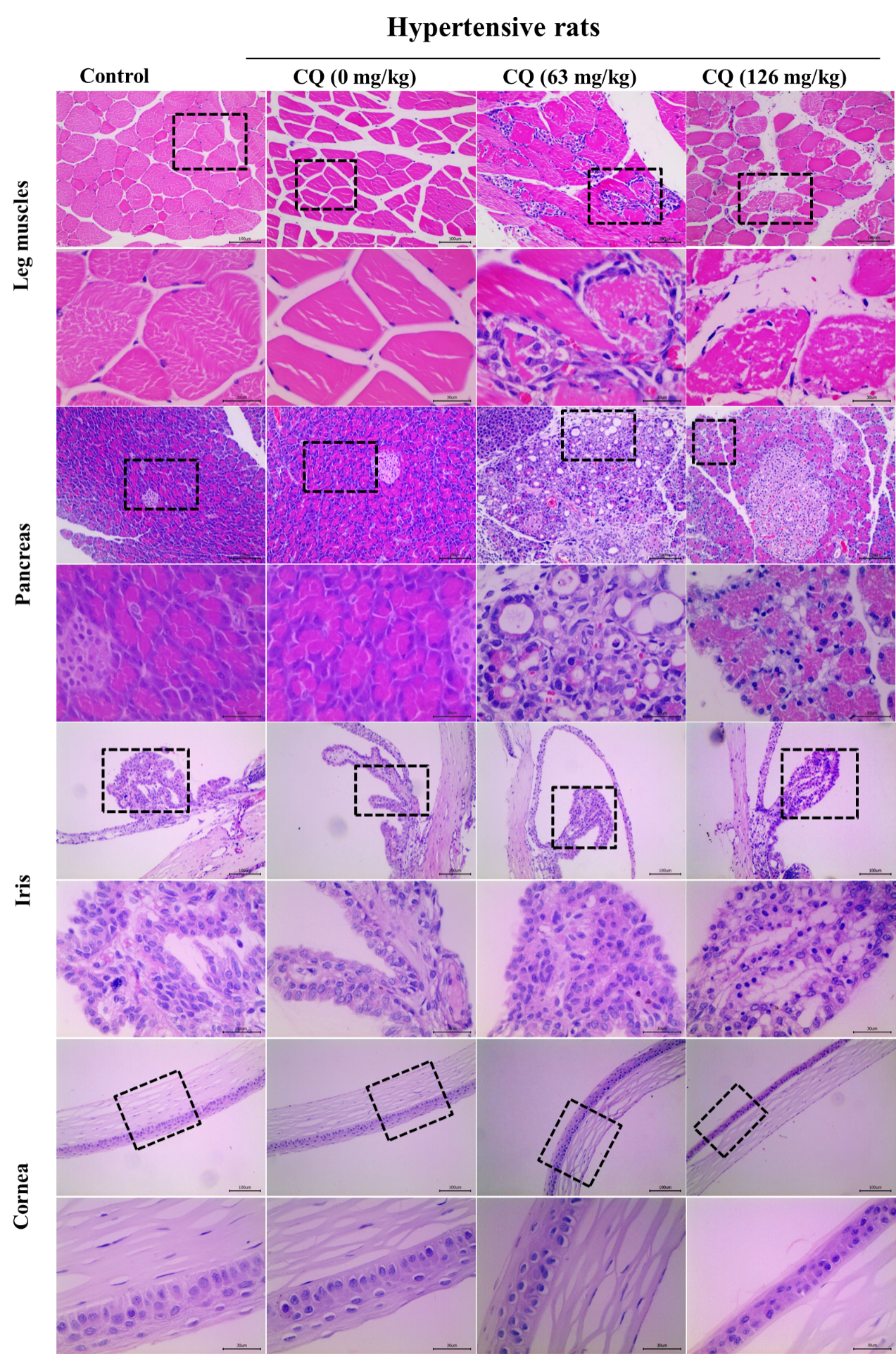


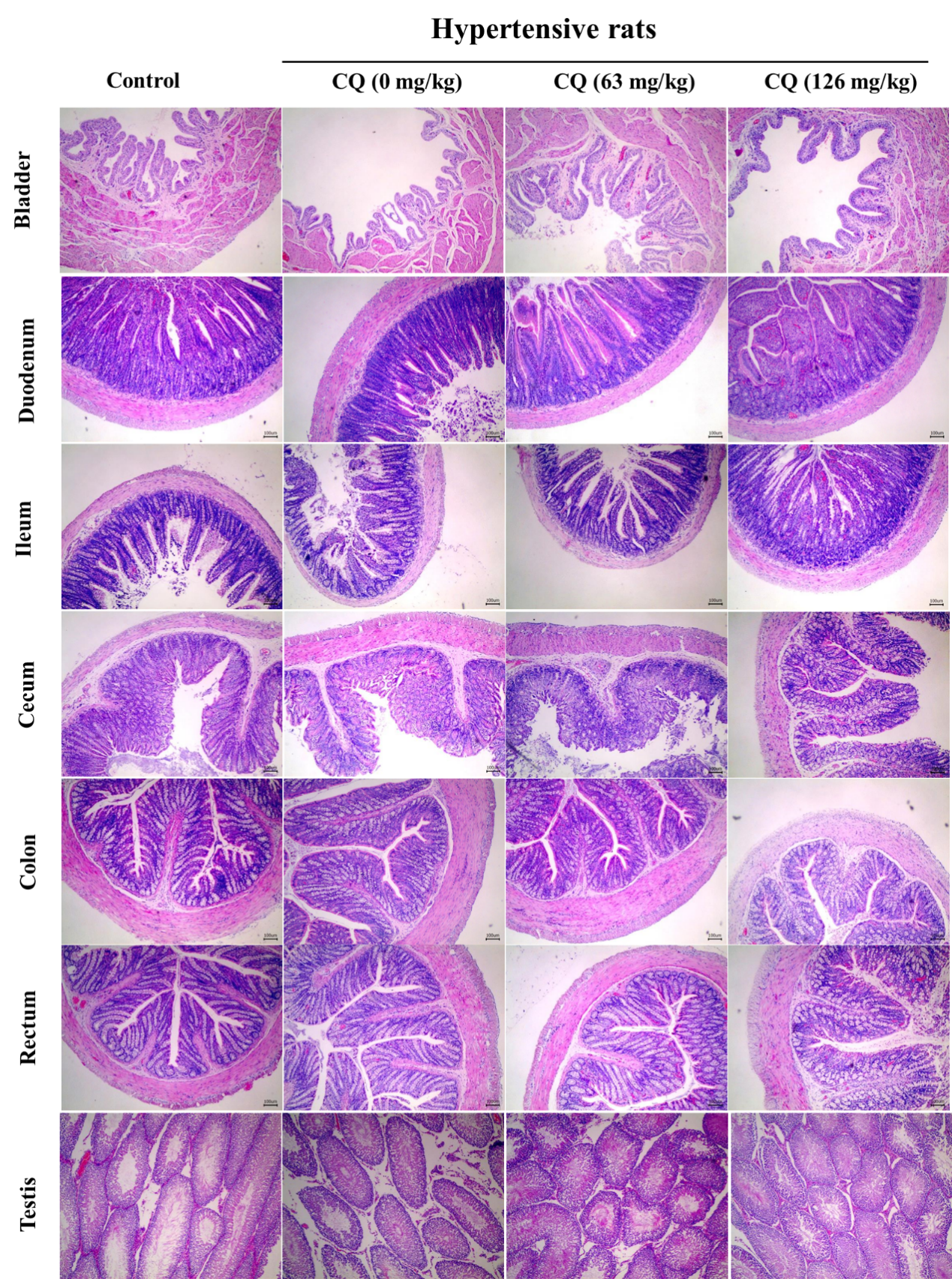


**Fig. S1** Histopathological changes of selected organs in chloroquine-treated rats

Supplement: Supplementary file 1 [file Table2.DOCX]
